# Relative validity of a non-quantitative 33-item dietary screener with a semi-quantitative food frequency questionnaire among young adults

**DOI:** 10.1017/jns.2023.57

**Published:** 2023-07-06

**Authors:** Lorentz Salvesen, Andrew K. Wills, Nina C. Øverby, Dagrun Engeset, Anine C. Medin

**Affiliations:** Department of Nutrition and Public Health, Faculty of Health and Sport Sciences, University of Agder, PO Box 422, Kristiansand 4604, Norway

**Keywords:** Aspects of diet quality, Diet quality score, Frequency of intake, Self-report, Relative validity

## Abstract

The objective of the study was to assess the concordance and ranking ability of a non-quantitative 33-item dietary screener developed to assess the diet of young adults in Norway, ‘MyFoodMonth 1.1’, compared to a semi-quantitative food frequency questionnaire (FFQ). Data were collected in a cross-sectional dietary survey evaluating the diets of students at the University of Agder, in southern Norway. The students were asked to complete both a dietary screener and an FFQ. Data collection was carried out from September to December 2020. Participants were first-year university students aged ≥18 years familiar with Scandinavian language. Almost half of the eligible sample (*n* 344) was excluded due to not completing the FFQ, compared to 1⋅7 % not completing the dietary screener, resulting in 172 (66 % female) participants with a median age of 21 years. For most items of the dietary screener (*n* 27/33, 82 %), all aspects of diet quality and components of the Diet Quality Score showed moderate-to-strong concordance with the FFQ evaluated using Kendall's tau-b analyses (*t* > 0⋅31), supported by visual inspection of box and whisker plots and descriptive ranking ability in a cross-tabulation. There was little evidence to suggest that concordance was dependent on sex. The concordance and ranking ability of ‘MyFoodMonth 1.1’ is considered satisfactory compared to a semi-quantitative FFQ. This rapid dietary assessment instrument presents a valuable addition to traditional instruments and a possible solution to recruit hard-to-reach parts of the population.

## Introduction

The requirement to complete long dietary questionnaires in nutritional studies is a threat to recruitment, representativeness and retention. Such studies are often overrepresented by women and motivated individuals from higher socio-economic groups^(1)^, while men, adolescents, young adults and individuals from lower socio-economic groups are often underrepresented and difficult to recruit^(2,3)^. Compared to food frequency questionnaires (FFQs) and other comprehensive dietary assessment methods, non-quantitative dietary screeners offer a way to reduce participant burden and potentially increase participation rate and reach into populations that are challenging to recruit.

Dietary screeners compromise detailed dietary information in favour of a simplified dietary assessment. They are short FFQs, often without portion sizes, designed to quickly (<15 min) assess the usual (long-term) intake of selected foods or food groups and aspects of diet quality in a population^(4)^. The overall trend in dietary assessment is shifting towards digital methods^(5,6)^, but there is limited information on whether dietary screeners are primarily digital or paper-based. Non-quantitative dietary screeners alone can be used to describe dietary intake, examine associations between diet and other variables and examine the effects of an intervention^(7)^.

Validation of dietary assessment instruments is important to assess whether the instrument measures what it purports to measure. Full-length FFQs^(8,9)^ and shorter FFQs^(10,11)^ have been developed, validated, and used in a Norwegian setting. The shorter FFQs, designed as semi-quantitative questionnaires assessing both frequency of intake and quantity of >60 food items, may not be considered as dietary screeners due to their length and complexity. To our knowledge, presently there is no validated non-quantitative dietary screener used for dietary assessment in Norway.

The overall aim of the study was to assess the relative validity of the non-quantitative dietary screener ‘MyFoodMonth 1.1’ using a semi-quantitative FFQ as the comparison. Specifically, our objective was to assess the concordance and ranking ability of ‘MyFoodMonth 1.1’ for all single food items in the dietary screener; aspects of diet quality (in the form of selected food group categories, and iodine and calcium intake); a Diet Quality Score (DQS); and to assess whether there was a difference in ranking abilities in those above by sex.

## Methods

### Study design and sample

Data are from StudentKost2 – a dietary survey of students at the University of Adger (UiA), Southern Norway (unpublished work). The purpose of StudentKost2 was to assess students’ diets in 2020, and to enable comparison between methods (dietary screener, 24 h dietary recall (24HR) and FFQ). Participants were recruited from August to October 2020. The recruitment strategies used were email, posters, flyers, videos in communal areas on campus, social media and in-person recruitment in classrooms. The target population was the 5003 first-year students aged ≥18 years at UiA familiar with Scandinavian language. A lottery of two iPhone 11s was used as an incentive to recruit participants.

**Fig. 1.**
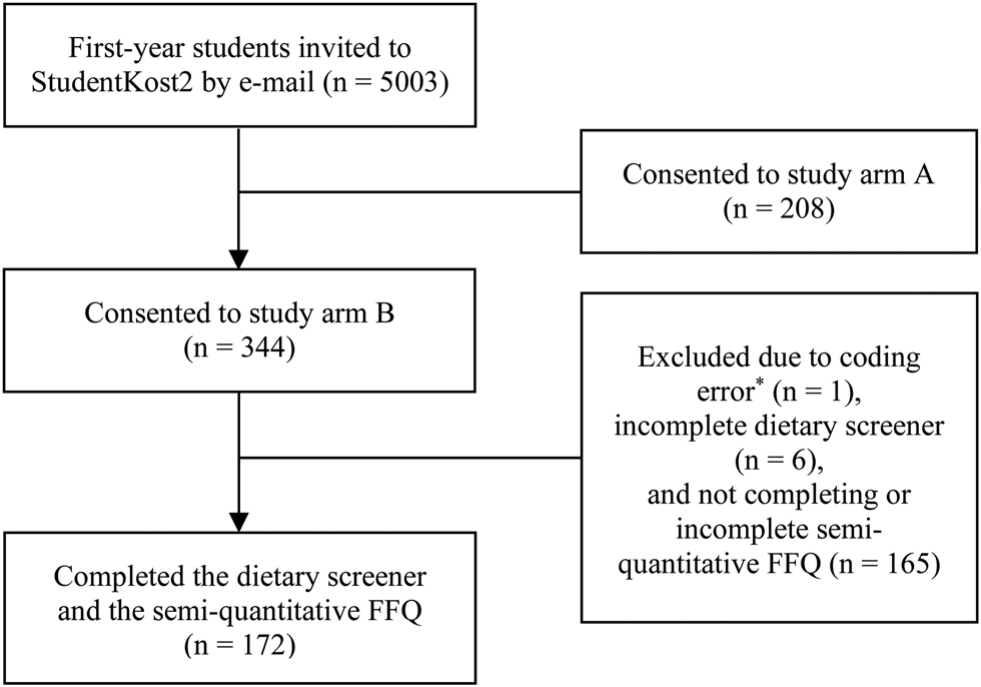
Flowchart for StudentKost2, study arm B. *Participant lost due to incorrect ID-number.

Participants could choose to participate in study arm A: complete a dietary screener and 2 × 24HR, or arm B: complete a dietary screener, 2 × 24HR and an FFQ. Participants in study arm B were randomly assigned to receive either the FFQ or the 24HR within 48 h after completing the dietary screener. Participants who were assigned to receive the 24HR first, were sent the FFQ at the earliest 5 weeks after completing the dietary screener. The FFQ used in study arm B was the same instrument as used in a previous dietary survey among students, StudentKost1^(12)^. Informed consent was collected electronically by individuals who actively choose to sign up for study arm A or B. The background information form (age, height, weight, body mass index (BMI) and parental education), the dietary screener and the FFQ were all electronically self-reported using a smartphone or a computer at a time of the participants’ choosing. Data used in the present study are from participants in study arm B. Fig. 1 presents the recruitment flowchart for study arm B, resulting in a total of 172 participants recruited (114 female, 58 male).

### ‘MyFoodMonth 1.1’ dietary screener

The dietary screener ‘MyFoodMonth 1.1’ was developed by the Lifecourse Nutrition research group at UiA. It assesses the intake of thirty-three food items for the previous month (30 d) using ten frequencies of intake ranging from ‘never’ to ‘6+ times per day’ (see Supplementary file 1). The dietary screener was based on the 2015 Dietary Screener Questionnaire from the National Cancer Institute's National Health Interview Survey Cancer Control Supplement^(13)^. The food list was altered to fit the Norwegian food culture, capturing intakes of fruit and vegetables, dairy, calcium, added sugars, whole grains/fibre, red meat and processed meat. All food items included were evaluated relative to data collected in a national dietary survey conducted among adults in Norway, the Norkost 2011 study^(14)^.

The dietary screener does not assess portion sizes and does not aim to assess diet in its entirety, nor to estimate energy intake or absolutes of macro- or micronutrients of foods. It is designed to assess frequency of intake of selected food groups and hence assess aspects of diet quality. Furthermore, the dietary screener is designed to rank individuals according to their intake of food items, food groups reflecting aspects of diet quality, and calcium and iodine intake. Consequently, the dietary screener is designed to discriminate between low and high intakes based on the ten frequency of intake categories.

Following the thirty-three food items, the dietary screener includes questions on dietary patterns and preferences: use of dietary supplements (if yes, then which and how often), frequency of meals per week (breakfast, lunch, dinner, supper and snack) and abstaining from certain foods and beverages (seven predefined options and two open-ended options). Finally, an open-ended question with the option to leave a comment related to the diet.

### Semi-quantitative FFQ

The FFQ^(15)^ used in the present study was also developed by the Lifecourse Nutrition group at UiA and aims to assess the diet of preconception young adults. The FFQ is based on a questionnaire used among adolescents in the Norwegian Mother, Father and Child Cohort Study^(9)^. It consists of 121 food items, assessing an estimate of habitual dietary intake 4 weeks in retrospect. The nutritional calculation and estimation of gram intakes are based on standard portion sizes and nutritional values from the Norwegian Food Composition Table^(16)^.

### Data cleaning

Dietary screener data were checked for coding errors in ID-number, incomplete data and suspicious registrations (defined *a priori* as individuals reporting the same frequency of intake for twenty-two or more and seventeen or more food items (66 and 50 % of food items, respectively)). The calculation of the nutrient and food intake from the FFQ is described elsewhere^(15)^. Individuals with incomplete FFQ recordings were excluded, as complete dietary data was required to calculate nutrient and food intake.

### Variables

Three data processing approaches were used to assess the relative validity of the dietary screener: first, keeping frequency categories as measured, i.e. raw measures; second, pooling food items and collapsing frequency categories to reflect aspects of diet quality; and third, as a DQS.

#### Harmonisation of dietary items between dietary screener and FFQ

Ninety-eight FFQ food items were aggregated into thirty-one groups corresponding to the food items in the dietary screener. Dietary screener food items ‘Plant-based meat substitutes’ and ‘Nuts and seeds, unsalted’ were not assessed by the FFQ and therefore not included in the comparison (Table 1). Food items in the FFQ that the author (LS) was unsure of whether to include in an aggregated group were discussed with a dietary expert (ACM) and solved by author consensus (LS, ACM) (Supplementary file 2).

**Table 1.** Description of the semi-quantitative food frequency questionnaire food items aggregated into the corresponding dietary screener food items

Dietary screener items	Semi-quantitative FFQ items
Cereal and porridge, sweetened (e.g. Special K, corn flakes with honey)	Cornflakes, All-Bran, Special K, Cheerios Oat Crunch or the like
Cereal and porridge, unsweetened (e.g. 4-Korn muesli, oatmeal, Go'dag muesli, and Weetabix)	Oatmeal/oat porridge
	Muesli
Whole-grain bread, crispbread, rolls (>50% whole grain)	Whole wheat bread/wheat bread, bread with a medium fibre content
	Crispbread, high fibre content
Fish spread (e.g. mackerel in tomato sauce)	Roe
	Fish spread or cold cuts
White cheese (all types)	White/yellow cheese
Whey cheese	Brown cheese
Yoghurt, skyr (all types)	Natural yoghurt
	Fruit yoghurt/drinking yoghurt, ordinary
	Fruit yoghurt/drinking yoghurt, sugar free/reduced sugar content
Cow's milk (all types)	Whole milk (sweet/sour, e.g. Kefir)
	Low-fat milk
	Extra skimmed milk
	Skimmed milk
	Cultured milk products (e.g. Biola)
	Chocolate milk
	Activia/Actimel drinking yoghurt
Plant-based milk (all types)	Soy milk, rice milk or other type of milk
Juice/smoothie (not nectar)	Orange juice
	Apple juice
Fruit and berries, including fresh, frozen and canned (not juice or smoothie)	Apple
	Pear
	Banana
	Orange, mandarin, clementine, grapefruit
	Nectarine, peach or plum
	Melon
	Kiwi
	Pineapple, fresh
	Berries, fresh or frozen
	Grapes
	Raisins
	Dried fruit
Unsalted nuts and seeds	N/A
Vegetables, including salad, cabbage, carrot, green beans, etc. (not potatoes or sweet potatoes)	Broccoli
	Cauliflower
	Onion, garlic or leek
	Avocado
	Maize
	Mushrooms
	Peas
	Mixed salad
	Spinach
	Green, yellow, orange or red pepper
	Carrots
	Cucumber
	Tomato
Beans, lentils, chickpeas, peas (not green beans)	Dishes with beans, lentils or peas
Fried potatoes/sweet potatoes (e.g. fries, roast potatoes)	French fries
Potatoes/sweet potatoes, other (e.g. baked, boiled, mashed)	Potatoes, cooked or mashed
Whole-grain dinner products (e.g. barley, pasta, couscous)	Rice, whole grain
	Pasta/spaghetti, whole grain
	Noodles, whole grain
Pizza (all types)	Pizza
Tomato sauce, including sauce/salsa for tacos, ketchup, pasta, etc. (not pizza)	Ketchup
Plant-based substitutes (all types of meat substitutes)	N/A
Red meat, minced or cuts (beef, lamb/mutton, pork, kid)	Pork
	Beef, lamb
Processed meat (e.g. bacon, spread, sausage)	Liver pâté
	Ham, roast beef or the like
	Salami, boiled sausage slices, cured meats or the like
	Chicken or turkey cold cuts
	Meatballs/patties
	Sausages (of pork and/or beef)
	Taco (tacos or mince wraps)
	Hamburger
	Casserole dish
	Pasta dish with meat
	Processed chicken products
Fatty fish and fish products (e.g. salmon, mackerel)	Oily fish
Lean fish and fish products (e.g. cod, pollock)	White fish
	Processed fish meat
Salty snacks (e.g. popcorn, chips, salty nuts)	Potato chips, tortilla chips
	Popcorn
	Nuts
Candy, including chocolate	Candy
	Vanilla and/or milk chocolate
	Dark chocolate
	Chocolate bar
Waffles, buns, cake, biscuits, etc.	Pie
	Pastries
	Cake
	Cookies
Ice cream, panna cotta, pudding, mousse, etc.	Ice cream
	Ice pop
	Pudding, mousse, jelly
	Rice pudding and rice cream dessert
	Canned fruit
	Custard
Sugar-sweetened beverages	Squash, sugar-sweetened (e.g. lemonade, Ribena)
	Other juice or nectar (e.g. tropical juice, breakfast juice)
	Soft drinks (e.g. Coca Cola, Fanta, Sprite)
Sugar-sweetened energy drinks (e.g. Gatorade, Red Bull)	Energy drinks (e.g. Red Bull, Battery, Pure Rush, Cult, Burn)
Coffee/tea/iced coffee/iced tea with sugar/syrup/honey	Frappuccino, mocaccino, ice coffee or the like
Alcoholic beverages	Beer
	Cider
	Wine
	Liquor, liqueur
Water	Tap water, bottled water or mineral water

Semi-quantitative FFQ, semi-quantitative food frequency questionnaire; N/A, not applicable.

#### Aspects of diet quality and nutrient intake

Single food items from the dietary screener were pooled to reflect aspects of diet quality (Table 2). The same pooling was used for the aggregated groups of the FFQ to sum up the intakes in grams. Dietary screener food items pooled to reflect the iodine and calcium intakes were compared with the total calculated nutrient intake of iodine and calcium from the FFQ. Simplified sets of pooled ordinal variables were derived to capture these aspects of diet quality components. The categories were chosen to (a) reflect typical intake and dietary guidelines in the Norwegian population^(14,17)^ and (b) ensure that certain categories did not have low cell counts (Table 2). For example, to reflect intake relative to guidelines, food items were recoded into categories from ‘<1 a day’ to ‘≥5 a day’ for fruit and vegetable intake, ‘<3⋅5 a week’ to ‘>2 a day’ for whole grain, ‘never’ to ‘≥2⋅5 a week’ for fish intake and ‘<1 a day’ to ‘≥5 a day’ for iodine-rich and calcium-rich foods. Red and processed meat, sugary foods, sugar-sweetened beverages, and beans, lentils, chickpeas, peas were recorded to present an even distribution of participants in categories ranging from ‘never’ to ‘≥1 a day’. For alcohol intake, the following categories were used: ‘never’ to ‘≥3⋅5 a week’.

**Table 2.** Description of the dietary screener food items derived into aspects of diet quality with collapsed frequency of intake and the Diet Quality Score with scoring valence

		Aspects of diet quality			Diet Quality Score	
		Dietary aspects		Frequencies of intake	Components	Scoring valence*
Dietary screener	Cereal and porridge (unsweetened)	Whole grain		<3⋅5 a week 3⋅5 a week to <1 a day 1–2 a day >2 a day	Whole grain (products)	Positive
	Whole-grain bread					
	Whole grain (barley/pasta/couscous)					
	Beans/lentils/chickpeas/peas	Beans, lentils, chickpeas, peas (not green beans)		Never >0 to <weekly Weekly to <3⋅5 a week 3⋅5 a week to <1 a day ≥1 a day	Beans and lentils	Positive
	Red meat (beef/lamb/pig/goat)	Red and processed meat		Never >0 to <weekly Weekly to <3⋅5 a week 3⋅5 a week to <1 a day ≥1 a day	Meat (processed and red)	Negative
	Processed meat					
	Cereal and porridge (sweetened)	Sugary foods		Never >0 to <weekly Weekly to <3⋅5 a week 3⋅5 a week to <1 a day ≥1 a day	Sugary foods	Negative
	Candy/chocolate					
	Waffles/buns/cake/biscuits					
	Ice cream/panna cotta/pudding, etc.					
	Sugar-sweetened beverages	Sugar-sweetened beverages		Never >0 to <weekly Weekly to <3⋅5 a week 3⋅5 a week to <1 a day ≥1 a day	Sugar-sweetened beverages	Negative
	Energy drinks with sugar					
	Fruit/berries (not juice/smoothie)	Fruit and vegetable		<1 a day 1–2⋅5 a day>2⋅5 to <5 a day ≥5 a day	Fruit	Positive
	Vegetables (not potatoes)				Vegetables	Positive
	Alcoholic beverages	Alcoholic beverages		Never >0 to <2 a month 2 a month to <weekly Weekly to <3⋅5 a week ≥3⋅5 a week	N/A	
	Nuts/seeds (unsalted)^†^	N/A			Nuts and seeds (unsalted)	Positive
	Salty snacks	Single ordinal variable		Never >0 to <2 a month 2 a month to <weekly Weekly to <3⋅5 a week 3⋅5 a week or more	Salty foods	Negative
	Fish spread	Fish		Never >0 to <1 a week 1 to <2⋅5 a week ≥2⋅5 a week	Fish	Positive
	Fatty fish (salmon/mackerel)					
	Lean fish (cod, pollock)					
	White cheese	Calcium-rich foods^§^	Iodine-rich foods^‡^	<1 a day 1–2⋅5 a day>2⋅5 to <5 a day ≥5 a day	N/A	
	Whey cheese					
	Yoghurt					
	Cow's milk					
	Plant-based milk	Single ordinal variable		Never >0 to <weekly Weekly to <3⋅5 a week 3⋅5 a week to <1 a day ≥1 a day	N/A	
	Juice/smoothie	Single ordinal variable		Never >0 to <2 a month 2 a month to <weekly Weekly to <3⋅5 a week 3⋅5 a week or more	N/A	
	Coffee/tea with sugar/syrup/honey	Single ordinal variable			N/A	
	Fried potatoes/sweet potatoes	Single ordinal variable			N/A	
	Potatoes/sweet potatoes (other)	Single ordinal variable			N/A	
	Pizza	Single ordinal variable			N/A	
	Tomato sauce	Single ordinal variable			N/A	
	Plant-based meat substitutes^†^	N/A			N/A	
	Water	Single ordinal variable		<1 a day 1–2⋅5 a day >2⋅5 to <5 a day ≥5 a day	N/A	

N/A, not applicable.

* Scoring valence: positive, scored 0–10 points from the lowest to the highest diet quality; negative, scored 10–0 points from the lowest to the highest diet quality.

† Not available for comparison with the semi-quantitative food frequency questionnaire (FFQ).

‡ Compared to total iodine intake (μg) per day calculated from the semi-quantitative FFQ.

§ Compared to total calcium intake (mg) per day calculated from the semi-quantitative FFQ.

### Diet Quality Score

A DQS was devised that closely resembles the WELL Diet Score^(18)^. Nutritional professionals in the WELL Diet Score project distributed 0–10 points weighted relative to the health benefits associated with the ten frequencies of intake for the individual DQS components. The same scoring system was used in the present study for all frequencies of intake except two categories. That is, in our study ‘1/week’ was equivalent to ‘1–2/week’, and ‘2–4/week’ was equivalent to ‘3–4/week’, e.g. the score of two points in the WELL Diet score for Vegetables ‘1–2/week’ was used for ‘1/week’ in our study (detailed scoring system of the DQS components is available in Supplementary file 3).

The ten DQS components ‘vegetables’, ‘fruits’, ‘whole grain (products)’, ‘beans and lentils’, ‘fish’, ‘nuts and seeds (unsalted)’, ‘sugar-sweetened beverages’, ‘sugary foods’, ‘meat (processed and red)’ and ‘salty foods’ were derived from nineteen dietary screener food items as described in Table 2. The latter four DQS components above were inversely scored. All DQS components but ‘nuts and seeds (unsalted)’ were available for comparison with the FFQ. As previously described, the dietary screener food item ‘nuts and seeds, unsalted’ was not assessed by the FFQ, hence not available for comparison in the DQS.

### Statistical analyses

Descriptive data for age, height, weight, BMI and parental education level were presented for the total sample and split by sex.

The dietary screener frequencies of intake were compared to FFQ data, both as intakes in grams, and nutrients. This was done to evaluate the non-quantitative dietary screener ability to reflect dietary intake without assigning portion sizes that would have correlated errors with the portion sizes in the FFQ.

Kendall's tau-b correlation analysis with bootstrap 95 % confidence intervals was used to estimate the concordance between raw measures from the dietary screener and the FFQ. A similar analysis was performed for the aspects of diet quality and DQS. Kendall's tau-b correlation coefficients were interpreted as follows: <0⋅30 = weak, 0⋅31–0⋅60 = moderate and >0⋅61 = strong^(19–21)^. We also cross-tabulated aspects of diet quality as ascertained from the dietary screener and FFQ to visually evaluate the ranking ability by comparing frequency of intake (assessed by the dietary screener) with median (IQR) (grams/nutrients per day) intake from the FFQ.

Box and whisker plots with participants as individual data points were produced to visualise the ranking ability using the raw measures from the thirty-one dietary screener food items available for comparison with the FFQ (grams per day). Participants were presented pooled, the box indicating median and IQR (25th and 75th percentiles), and whiskers using the quartile ±1⋅5*IQR convention.

Kendall's tau was repeated using *a priori* cut-offs as sensitivity analyses after removing individuals who reported the same frequency of intake for twenty-two or more and seventeen or more food items in the dietary screener. We suspected that these individuals were only interested in receiving the incentive and likely gave the same easy click response to each question to save time.

Kendall's tau analyses were performed using SPSS 25 (IBM Corp. Released 2017. IBM SPSS Statistics for Windows, Version 25.0. Armonk, NY: IBM Corp.), plots were produced in STATA (v17.0) and R (v 2022.2.0.443).

### Ethical standards

This study was conducted according to the guidelines laid down in the Declaration of Helsinki and all procedures involving research study participants were approved by the Norwegian Centre for Research Data (ref.nr: 848472) and the ethical committee for the Faculty of Health and Sport Science at the University of Agder (ref.nr: RITM0070447). Informed consent by action was obtained electronically from all subjects.

## Results

### Sample description

Fig. 1 shows that of the 344 participants eligible for inclusion, 1⋅7 % were excluded due to incomplete dietary screener submission, and 48 % due to incomplete or non-completion of the FFQ. Table 3 describes the characteristics of the 172 participants who completed both the dietary screener and FFQ. The median age was 21 years and 66 % were females. The median BMI was within the healthy weight range (18⋅5–24⋅9 kg/m^2^)^(22)^, and most of the participants had one or two parents who had completed higher education.

**Table 3.** Descriptive statistics for participants completing both the dietary screener and the semi-quantitative food frequency questionnaire, presented as median with interquartile range and frequency with proportion, unless stated otherwise

	Total		Female		Male	
	(*n* 172)		(*n* 114)		(*n* 58)	
	Median	IQR	Median	IQR	Median	IQR
Age, years	21	(19, 25)	21	(19, 24)	22	(20, 25)
Height, cm*	173	(9)	168	(6)	181	(8)
Weight, kg	68^†^	(60, 79)	65^‡^	(58, 73)	78^§^	(69, 90)
BMI	23^†^	(20⋅9, 25⋅7)	22⋅8^‡^	(20⋅5, 25⋅6)	23⋅4^§^	(21⋅6, 26⋅2)
Parental education level, *n* (%)						
Lower education	37	(22%)	28	(25%)	9	(16%)
Vocational secondary school	39⋅5	(23%)	26⋅5	(23%)	13	(22%)
Higher education	85	(49%)	53⋅5	(47%)	31⋅5	(54%)
Other	10⋅5	(6%)	6	(5%)	4⋅5	(8%)

IQR, interquartile range; sd, standard deviation; BMI, body mass index.

* Presented as mean (sd). Reporting weight was optional, resulting in sample variation for weight and BMI.

† *n* 157.

‡ *n* 101.

§ *n* 56. BMI calculated as kg/m^2^. Parental education level: Lower education (primary school and secondary school), Higher education (university, less than 4 years and university, more than 4 years), and Other (other education and not sure/not applicable).

### Concordance between the dietary screener and the semi-quantitative FFQ food items

The concordance quantified using Kendall's tau between the raw measures from the dietary screener and the thirty-one food items available for comparison with the FFQ (grams per week) are plotted in Fig. 2. These ranged from 0⋅20 (95 % CI 0⋅07–0⋅31) (weak concordance) for tomato sauce (including sauce/salsa for tacos, ketchup, pasta, etc., but not pizza) to 0⋅79 (95 % CI 0⋅71–0⋅86) (strong concordance) for whey cheese. Twenty food items (65 %) had concordance between 0⋅31 and 0⋅60 (moderate) and seven food items (23 %) greater than 0⋅61 (strong). The raw measures from the dietary screener and grams per day from the FFQ are also visually presented as box and whisker plots (available in Supplementary file 4, Figs. S1–S31).

**Fig. 2.**
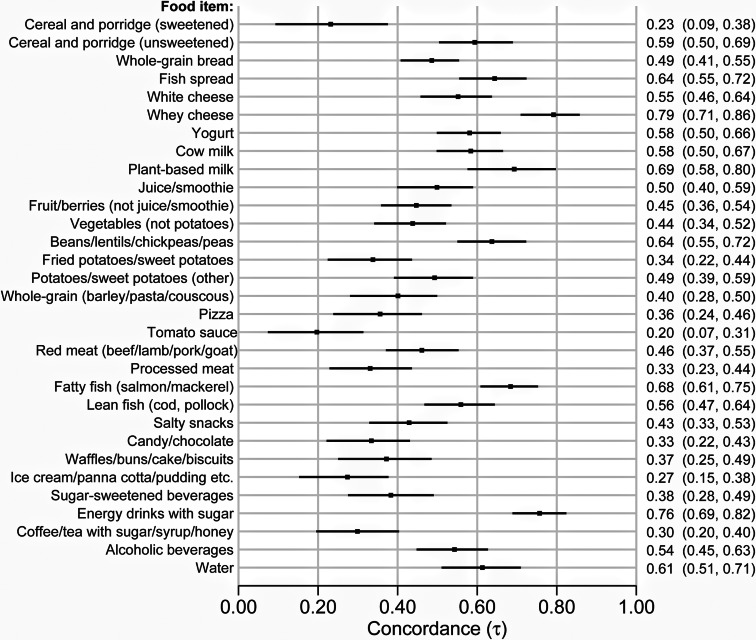
Forest plot of Kendall's tau-b concordance with 95 % confidence intervals for thirty-one food items in the dietary screener ‘MyFoodMonth 1.1’ compared to a semi-quantitative food frequency questionnaire.

The concordance of the food items in the dietary screener split by sex is available in Supplementary file 5, Fig. S32. The concordance was generally similar between the sexes – only five out of thirty-one food items showed any suggestion of difference. For female participants, the concordance varied from 0⋅13 (95 % CI −0⋅03 to 0⋅031) for cereal and porridge, sweetened (e.g. Special K) to 0⋅78 (95 % CI 0⋅69–86) for whey cheese. For male participants, the concordance varied from 0⋅18 (95 % CI 0⋅00–0⋅36) for coffee/tea/iced coffee/iced tea with sugar/syrup/honey to 0⋅84 (95 % CI 0⋅65–0⋅99) for whey cheese. The greatest differences in concordance between female and male participants were observed for plant-based milk (all types) (0⋅74 (95 % CI 0⋅62–0⋅85) and 0⋅45 (95 % CI 0⋅14–0⋅72), respectively), red meat, minced or cuts (beef, lamb, pork, goat) (0⋅51 (95 % CI 0⋅40–0⋅62) and 0⋅22 (95 % CI 0⋅02–0⋅42)), and cereal and porridge, sweetened (e.g. Special K) (0⋅13 (95 % CI −0⋅03 to 0⋅31) and 0⋅43 (95 % CI 0⋅17–0⋅67)).

There were no individuals who reported a suspicious sequence of the same frequency of intake for twenty-two or more food items in the dietary screener. Only four individuals reported a suspicious sequence of the same frequency of intake for seventeen or more food items. In a sensitivity analysis removing these individuals, results were unaltered.

### Aspects of diet quality and DQS

Table 4 reports the distribution of values and ranking of the FFQ and the DQS according to the aspects of diet quality defined using the dietary screener. A visual inspection of the median and IQR (25th and 75th percentiles) shows that the dietary screener distinguished between high and low intake for most variables. An unbalanced distribution of participants (mainly among males) for the aspects ‘fruit and vegetable’, ‘red and processed meat’ and ‘beans, lentils and chickpeas’ presented in Table 4 affect the credibility of the estimates.

**Table 4.** Cross-table of aspects of diet quality, including iodine and calcium intake, derived from the dietary screener ‘MyFoodMonth 1.1’ and the distribution of intakes from the semi-quantitative food frequency questionnaire, and the Diet Quality Score

			Female					Male				
			FFQ (g)			DQS (0–10)		FFQ (g)			DQS (0–10)	
	Aspects of diet quality	Frequency of intake	*N*	Median	(IQR)	Median	(IQR)	*n*	Median	(IQR)	Median	(IQR)
Dietary screener	Fruit and vegetable*	<1 a day	29	121	(71, 180)	6	(5, 8)	26	109	(49, 158)	4⋅5	(3, 8)
		1–2⋅5 a day	54	217	(140, 368)	12	(10, 14)	25	202	(166, 270)	10	(9, 12)
		>2⋅5 to <5 a day	19	193	(159, 295)	15	(12, 18)	6	236	(163, 347)	11	(10, 15)
		≥5 a day	12	447	(365, 568)	19	(19, 19)	1	568	(568, 568)	19	(19, 19)
	Whole grain	<3⋅5 a week	19	35	(9, 71)	1	(0, 4)	9	62	(15, 127)	2	(0, 4)
		3⋅5 a week to <1 a day	19	73	(30, 101)	4	(4, 6)	10	69	(36, 90)	6	(4, 6)
		1–2 a day	38	129	(73, 183)	8	(8, 8)	17	134	(66, 186)	8	(8, 8)
		>2 a day	38	174	(133, 221)	10	(10, 10)	22	173	(113, 218)	10	(10, 10)
	Fish	Never	14	0	(0, 0)	0	(0, 0)	15	0	(0, 0)	0	(0, 0)
		>0 to <1 a week	28	21	(11, 30)	7	(4⋅75, 7)	7	16	(13, 45)	10	(4, 10)
		1 to <2⋅5 a week	27	36	(27, 46)	10	(10, 10)	23	40	(31, 50)	10	(10, 10)
		≥2⋅5 a week	45	77	(51, 116)	10	(10, 10)	13	64	(36, 83)	10	(10, 10)
	Red and processed meat^†^	Never	11	0	(0, 7)	10	(10, 10)	2	53	(14, -)	10	(10, 10)
		>0 to<weekly	10	86	(34, 130)	9	(8, 10)	2	22	(11, -)	10	(10, 10)
		Weekly to <3⋅5 a week	26	78	(56, 101)	6	(6, 6)	12	138	(74, 214)	6	(4⋅5, 6)
		3⋅5 a week to <1 a day	45	127	(86, 178)	2	(2, 4)	27	162	(126, 258)	4	(2, 4)
		≥1 a day	22	130	(93, 251)	1	(0, 1)	15	233	(171, 283)	1	(0, 1)
	Sugary foods^†^	Never	1	12	(12, 12)	10	(10, 10)	1	8	(8, 8)	10	(10, 10)
		>0 to <weekly	14	11	(6, 29)	6	(6, 8)	9	16	(10, 28)	8	(6, 8)
		Weekly to <3⋅5 a week	40	32	(23, 39)	6	(6, 6)	24	35	(20, 53)	6	(6, 6)
		3⋅5 a week to <1 a day	34	43	(23, 61)	4	(1, 4)	18	38	(24, 55)	4	(4, 4)
		≥1 a day	25	45	(34, 75)	0	(0, 0)	6	56	(38, 78)	0	(0, 0)
	Sugar-sweetened beverages^†^	Never	21	0	(0, 36)	10	(10, 10)	9	36	(18, 143)	10	(10, 10)
		>0 to <weekly	47	50	(36, 71)	8	(8, 9)	11	50	(36, 114)	9	(6, 9)
		Weekly to <3⋅5 a week	13	100	(64, 243)	4	(4, 6)	12	79	(55, 184)	4	(4, 6)
		3⋅5 a week to <1 a day	21	143	(79, 357)	4	(1, 4)	14	179	(143, 314)	2⋅5	(1, 4)
		≥1 a day	12	175	(20, 461)	0	(0, 0)	12	386	(179, 712)	0	(0, 0)
	Beans, lentils, chickpeas, peas (not green beans)	Never	40	0	(0, 0)	0	(0, 0)	19	0	(0, 0)	0	(0, 0)
		>0 to <weekly	44	21	(0, 21)	2	(1⋅25, 3⋅5)	24	21	(21, 43)	2	(1, 4)
		Weekly to <3⋅5 a week	14	86	(37, 129)	6	(6, 6)	8	43	(21, 129)	6	(6, 6)
		3⋅5 a week to <1 a day	8	129	(27, 129)	8	(8, 8)	2	32	(21, −)	8	(8, 8)
		≥1 a day	8	129	(43, 193)	9	(9, 9)	5	43	(43, 161)	9	(9, 9)
	Alcoholic beverages	Never	27	0	(0, 0)	N/A		11	0	(0, 36)	N/A	
		>0 to <2 a month	21	79	(41, 138)			8	68	(36, 113)		
		2 a month to <weekly	53	95	(49, 206)			26	150	(76, 306)		
		Weekly to <3⋅5 a week	11	191	(60, 372)			12	477	(298, 676)		
		≥3⋅5 a week	2	514	(290, −)			1	1335	(1335, 1335)		
	Iodine-rich foods	<1 a day	31	86^‡^	(60, 119)	N/A		13	66^‡^	(55, 107)	N/A	
		1 to 2⋅5 a day	48	113^‡^	(69, 150)			27	106^‡^	(88, 142)		
		>2⋅5 to <5 a day	29	132^‡^	(93, 214)			14	160^‡^	(58, 205)		
		≥5 a day	6	311^‡^	(212, 460)			4	157^‡^	(150, 185)		
	Calcium-rich foods	<1 a day	35	535^§^	(324, 679)	N/A		14	616^§^	(410, 801)	N/A	
		1 to 2⋅5 a day	47	751^§^	(571, 905)			28	814^§^	(652, 971)		
		>2⋅5 to <5 a day	27	891^§^	(673, 1353)			12	1107^§^	(888, 1302)		
		≥5 a day	5	1828^§^	(1396, 2045)			4	1130^§^	(921, 1416)		

FFQ, semi-quantitative food frequency questionnaire; DQS, Diet Quality Score; IQR, interquartile range; N/A, not applicable.

* Diet quality score components ‘Vegetable’ and ‘Fruit’ combined, ranging from 0 to 20.

† Diet Quality Score component inversely scored.

‡ Compared to total iodine intake (μg).

§ Compared to total calcium intake (mg).

**Fig. 3.**
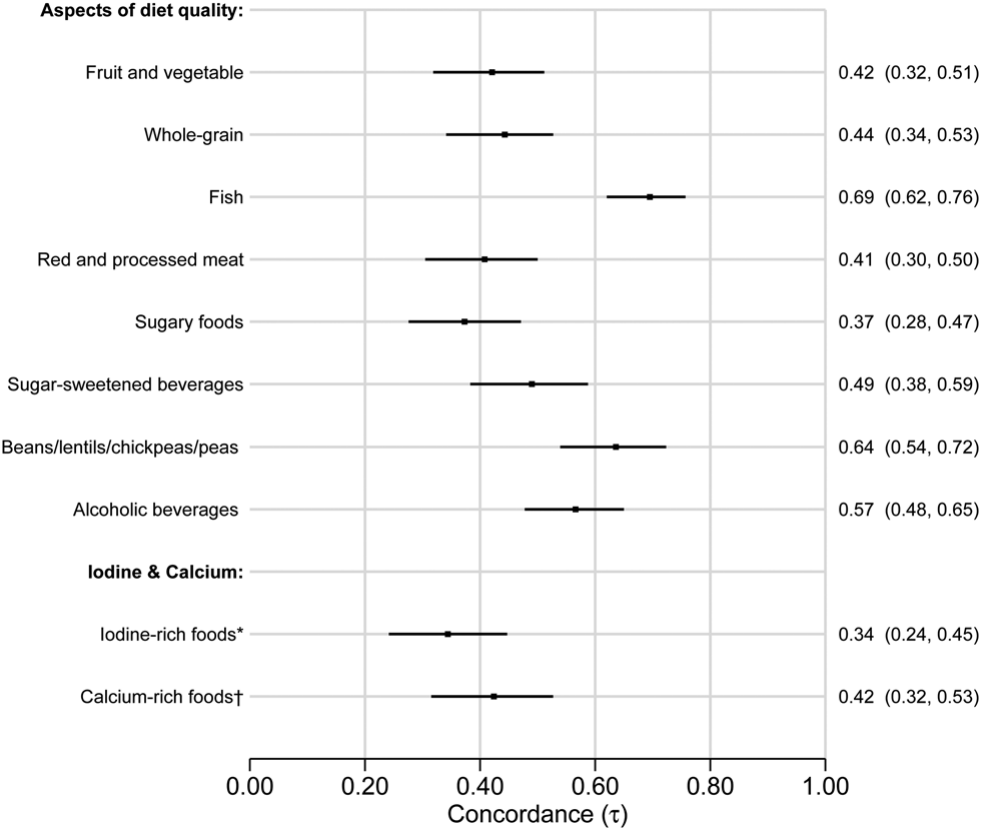
Forest plot of Kendall's tau-b concordance with 95 % confidence intervals for aspects of diet quality, iodine-rich foods and calcium-rich foods derived from the dietary screener ‘MyFoodMonth 1.1’ compared to a semi-quantitative food frequency questionnaire. *Compared to iodine intake (μg) per day. ^†^Compared to calcium intake (mg) per day.

Fig. 3 formally quantifies the concordance between the aspects of diet quality derived from the dietary screener (frequency of intake) and the FFQ (grams per day), ranging from 0⋅37 (95 % CI 0⋅28–0⋅47) for sugary foods to 0⋅70 (95 % CI 0⋅62–0⋅76) for fish. The ability of the dietary screener to capture intake of iodine and calcium (based on foods rich in these nutrients) compared to nutrient values in the FFQ showed moderate concordance of 0⋅34 (95 % CI 0⋅24–0⋅45) and 0⋅42 (95 % CI 0⋅32–0⋅53), respectively. Concordance for the nine single food items (ordinal variables) derived from the dietary screener, and that are not included as aspects of diet quality, ranged from 0⋅19 (95 % CI 0⋅06–0⋅30) for tomato sauce to 0⋅69 (95 % CI 0⋅59–0⋅79) for plant-based milk (available in Supplementary file 5, Fig. S33).

Concordance of the aspects of diet quality, iodine-rich foods and calcium-rich foods split by sex are available in Supplementary file 5, Fig. S34, and single food item ordinal variables that are not included as aspects of diet quality in Supplementary file 5, Fig. S35. The concordance was generally similar between the sexes – only two out of nineteen variables showed any suggestion of difference. The greatest differences in concordance between female and male participants for aspects of diet quality were observed for fruit and vegetable (0⋅37 (95 % CI 0⋅25–0⋅49) and 0⋅51 (95 % CI 0⋅36–0⋅64)) and alcoholic beverages (0⋅52 (95 % CI 0⋅40–0⋅63) and 0⋅65 (95 % CI 0⋅48–0⋅77)), and for the single food items (ordinal variables), the greatest differences were observed for plant-based milk (0⋅75 (95 % CI 0⋅62–0⋅85) and 0⋅45 (95 % CI 0⋅11–0⋅73)) and coffee/tea with sugar/syrup/honey (0⋅37 (95 % CI 0⋅25–0⋅49) and 0⋅18 (95 % CI −0⋅01 and 0⋅37)), respectively.

**Fig. 4.**
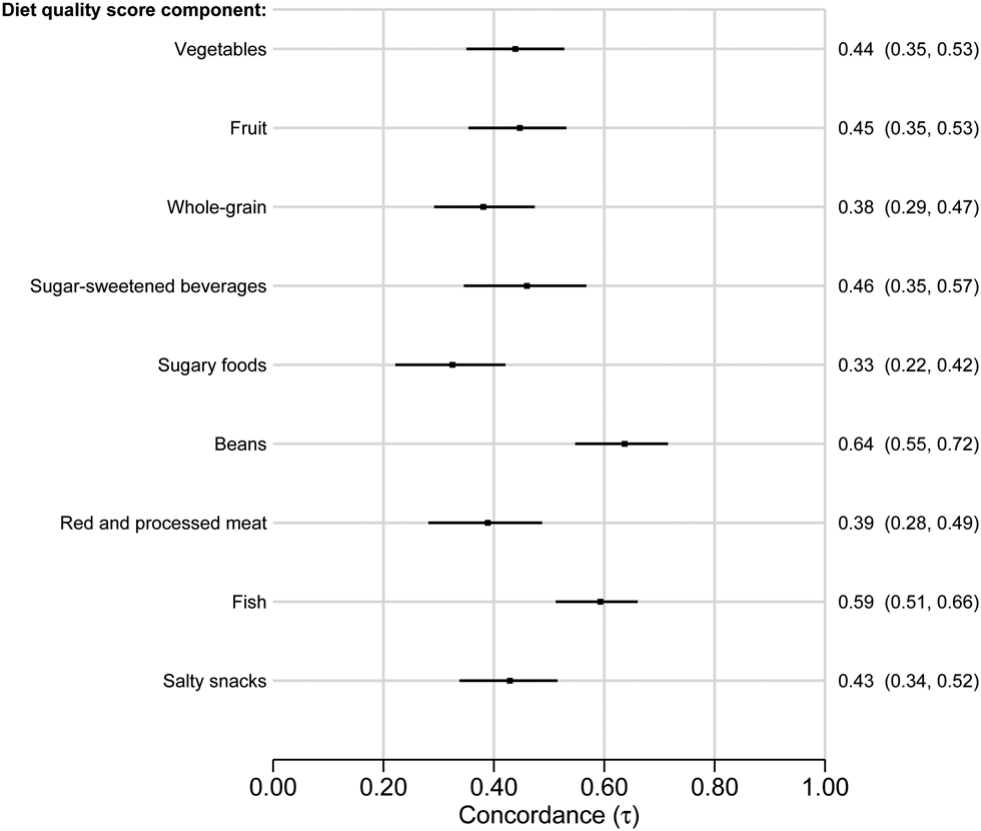
Forest plot of Kendall's tau-b concordance with 95 % confidence intervals for the Diet Quality Score components derived from the dietary screener ‘MyFoodMonth 1.1’ compared to a semi-quantitative food frequency questionnaire.

Fig. 4 presents Kendall's tau for the nine DQS components with available comparators in the FFQ. The concordance ranged from 0⋅33 (95 % CI 0⋅22–0⋅42) for sugary foods to 0⋅64 (95 % CI 0⋅55–0⋅72) for beans, lentils and chickpeas. The concordance split by sex is available in Supplementary file 5, Fig. S36. The concordance was generally similar between the sexes. The largest difference in concordance between female and male participants was observed for the DQS component vegetables (0⋅37 (95 % CI 0⋅24–0⋅49) and 0⋅50 (95 % CI 0⋅35–0⋅64)), respectively.

## Discussion

### Summary of findings

As far as we are aware, this is the first validation study of a non-quantitative dietary screener to assess dietary intake among young adults in a Norwegian population. The 33-item dietary screener was compared to a 121-food item FFQ. Kendall's tau-b analyses showed that twenty-seven of the thirty-one dietary screener variables available for comparison with the FFQ had a moderate or strong concordance (>0⋅31). The aspects of diet quality, and DQS, derived from the dietary screener, were all considered satisfactory. That is, they showed moderate-to-strong concordance with the FFQ. This was corroborated by the ranking ability visualised in a cross-table showing aspects of diet quality with DQS components and intakes from the FFQ. There was little evidence to suggest that concordance between the dietary screener and FFQ was dependent on sex.

### Comparison with other studies

Many validation studies on dietary assessment methods exist, but there are a limited number of validation studies on dietary screeners. Studies available for comparison have used a variety of validation approaches that are not directly comparable to ours.

### Fruit and vegetable intakes assessed with a dietary screener compared to an FFQ

The previously reported concordance for the intakes of fruit (0⋅42^(23)^, 0⋅61^(24)^, 0⋅63^(25)^, 0⋅54^(26)^) and vegetable (0⋅30^(23)^, 0⋅49^(24)^, 0⋅41^(25)^, 0⋅39^(26)^), separately, are comparable to our findings. Furthermore, our pooled fruit and vegetable variable as an aspect of diet quality is comparable to Dehghan *et al.*^(27)^ at 0⋅49, while Block *et al.*^(28)^ report a higher concordance at 0⋅71. The discrepancy between our results and those of Block *et al.* may be due to their use of seven questions to assess the intake of fruits and vegetables in the dietary screener compared to our use of two questions. This makes the dietary screener of Block *et al.* much more detailed and more like the FFQ they used in their comparison. Furthermore, in contrast to our study, Block *et al.* used defined portion sizes (small/medium/large) in their FFQ in addition to software to generate age- and gender-specific portion sizes, which may have yielded more accurate portion size estimations for comparison with their dietary screener.

### Fish and/or other seafood intakes assessed with a dietary screener compared to an FFQ

Our results for fish and seafood are also comparable to others (0⋅56 (oily fish)^(24)^, 0⋅68^(27)^, 0⋅46^(26)^), and substantially stronger compared to the concordance of Hebestreit *et al.*^(23)^ at 0⋅25. We speculate whether parts of the discrepancy between our results and those of Hebestreit *et al.* may be due to our dietary screener using three questions to assess fish intake, covering intake of different types of fish, compared to the single question (servings of fish/seafood per week) used in the study of Hebestreit *et al.*

### Red and processed meat intakes assessed with a dietary screener compared to an FFQ

Comparing our results with those of de Rijk *et al.*^(25)^ we found a similar concordance for red meat (0⋅30) and somewhat lower for processed meat (0⋅55). Our pooled variable for red and processed meat is comparable to Dehghan *et al.*^(27)^ (0⋅40), whereas Hebestreit *et al.*^(23)^ report a higher concordance of 0⋅58. Our non-quantitative dietary screener variables assessing red and processed meat intake is not designed to discriminate between portion sizes. Splitting these into more variables, enabling us to distinguish between intake of meat as cuts or meat for dinner (typically having very different portion sizes), could have contributed to a better concordance with the current FFQ.

### Sugar-sweetened beverages and sugary food intakes assessed with a dietary screener compared to an FFQ

Other studies report concordance for sugar-sweetened beverages between −0⋅04^(23)^ and 0⋅74^(27)^, indicating that it may be difficult to accurately assess. We found concordance in the area of 0⋅40 for sugar-sweetened beverages, indicating moderate concordance compared to the FFQ. For sugary foods, our results are comparable to other studies (0⋅44^(23)^, 0⋅39^(25)^). It should be noted that the single food item ‘cereal and porridge, sweetened’ included in the pooled variables defined as ‘sugary foods’ performed poorly compared to the FFQ, both in raking of participants intake (Supplementary file 4, Fig. S1) and for the concordance for female participants (Supplementary file 5, Fig. S36). We speculate that there are more women than men eating this kind of food and that the high concordance for men reflects the non-consumers. For females, it could be that they find it difficult to know if the cereal should be defined as sweetened or not – perhaps leading to misclassifications between the dietary screener and the FFQ. We suggest refining the variable ‘cereal and porridge, sweetened’ for future use of the dietary screener.

### Alcohol intake assessed with a dietary screener compared to an FFQ

We found somewhat stronger concordance for our total alcohol intake variable compared to other studies, showing concordance at 0⋅35 for wine^(23)^ and 0⋅41^(25)^ for pooled alcohol consumption. The concordance of individuals’ alcohol intake was assessed by comparing a single question in the dietary screener with the total alcohol intake reported for the week and weekend in the FFQ in the present study. This is similar to de Rijk *et al.*^(25)^, although they assessed intake split into week and weekend in the dietary screener, but used a single question in the FFQ. Our dietary screener shows surprisingly good concordance of participants’ intake of alcohol compared to de Rijk *et al.* We speculate that this may be due to the proportion of non-consumers in our study.

### Whole grain and legume intakes assessed with a dietary screener compared to an FFQ

The strength of the concordance for our pooled whole grain variable (‘cereal and porridge, unsweetened’, ‘whole-grain bread’ and ‘whole-grain dinner products’) is consistent with what has been reported in other studies for fairly comparable variables (0⋅35 (starches)^(27)^, 0⋅22^(25)^ (whole-grain products)). On the other hand, our findings for beans, lentils and chickpeas showed a considerably stronger concordance (0⋅64) than those found for legumes by de Rijk *et al.*^(25)^ at 0⋅43. We speculate that this discrepancy may be explained by the high number of non-consumers of beans, lentils and chickpeas observed in our study, because it is more difficult to report the correct intake of a food you eat sometimes or often than foods you never eat^(29)^. Nevertheless, the results show that there is a high concordance between (zero or higher) intakes of legumes in both the dietary screener and FFQ in our study.

### Calcium-rich foods and iodine-rich foods intakes assessed with a dietary screener compared to an FFQ

The only study that is comparable in some measure to ours, in regard to the calcium and iodine concordance, is by van Lee *et al.*^(26)^, who found an inverse association between their crude dietary screener index and estimated calcium intake from a full-length FFQ. However, this association disappeared when adjusted for energy intake estimates. In stark contrast, we found moderate concordance between the dietary screener intakes of calcium-rich and iodine-rich foods and the estimated total nutrient intake of calcium or iodine calculated from the FFQ. This shows that our non-quantitative dietary screener may provide a rough estimate of the level of calcium and iodine intake, despite not assessing the total diet or calculating nutrient intakes.

### Strengths and limitations

A strength of our study is the sample size, which enabled us to estimate the concordance with adequate precision^(30,31)^. We were also able to stratify our analysis by sex to check whether the dietary screener performs differently for males and females.

We also display the individual data points, which allows the concordance to be given a visual context and may be of use for future researchers who are interested in components of the dietary screener.

‘MyFoodMonth 1.1’ is purposively designed as a non-quantitative dietary screener. To estimate the quantities of foods from the dietary screener, we could have assigned standardised age- and gender appropriate portion sizes after data collection, as others have done previously, e.g. Block *et al.*^(28)^. We did not adopt this approach, because we wanted to avoid introducing additional estimation error, and a false impression of instrument resolution – using a ‘one-size-fits-all’ portion size (even adjusted for age- and gender, or body size, etc.) will not capture the between-person variation in portion sizes^(30)^. Moreover, by avoiding portion sizes, we strengthen the applicability of the instrument by reducing the time and resources necessary for data processing.

The sample population consists entirely of first-year students, limiting the generalisability. The student population with a 33 % proportion of men in our study is comparable to the student population at UiA^(32)^ and nationwide in Norway^(33)^ per 2021 (41 and 40 % men, respectively). However, the median BMI in our study sample is comparable to the mean BMI (24⋅2 for female, 23⋅8 for male) for young adults (18–24 years) reported in the Norwegian National Public Health Survey 2020^(34)^. Furthermore, the variation in parental education level in this study strengthens its generalisability to a general population of young adults in Norway.

### Implications

The non-quantitative dietary screener validated in this study is a rapid instrument assessing diet in a simple and effective way, with the potential to reach populations difficult to recruit using traditional dietary assessment instruments (e.g. FFQ and 24HR). As shown in Fig. 1, 48 % of participants eligible for inclusion did not complete the FFQ, whereas <2 % did not complete the dietary screener, illustrating this point.

This dietary screener may have utility as a main dietary assessment instrument, as a supplement to other dietary assessment instruments, or for studies with diet as a secondary outcome to reduce the total burden of the data collection.

The food items ‘cereal and porridge, sweetened’, ‘tomato sauce’ and ‘coffee/tea/iced coffee/iced tea with sugar/syrup/honey’ showed poor concordance with the FFQ. We suggest altering all three in future versions of the dietary screener.

‘Cereal and porridge, sweetened’ was included in both the aspect of diet quality component ‘sugary foods’, and the DQS component ‘sugary foods’, and should therefore ideally be kept in the dietary screener. To improve the ‘cereal and porridge, sweetened’ item in the dietary screener, we believe we need to clarify the difference between the sweetened and unsweetened cereal and porridge by altering the explanation texts for these food items. A suggestion would be to instruct participants to categorise cereals and porridge according to the ‘Keyhole’ scheme, a well-known^(35)^ label used in the Nordic Region based on the Nordic Nutrition Recommendations^(36)^, intended to make it easier for shoppers to choose better and healthier products. This could be done by simply adding the Keyhole label besides the unsweetened cereal and porridge food item. For cereals or breakfast cereals to carry the ‘Keyhole’ label, they must satisfy certain requirements: fat at most 8/100 g; sugars at most 13/100 g, of which added sugars at most 9/100 g; dietary fibre at least 6/100 g and salt at most 1/100 g^(37)^, which fits well with the ‘cereal and porridge, unsweetened’.

‘Coffee/tea/iced coffee/iced tea with sugar/syrup/honey’ may have performed poorly because it was too heterogeneous, comprising beverages with varying sugar content. Moreover, it was never included in either aspects of diet quality or a DQS component. This was because it comprises beverages with lower sugar content compared to other typical SSBs, e.g. two sugar cubes (4 g) in a small cup of coffee (100 g) compared to 10/100 g sugar content in regular soda. However, this food item category also includes iced tea, which often has sugar content similar to regular soda. Due to this, we suggest that in future versions of the dietary screener, we should include iced tea in the SSB-variable, and omit the lower sugar containing coffees and teas.

‘Tomato sauce’ comprising different kinds of tomato-based sauces, spanning from ketchup to e.g. a Bolognese sauce, was not included in either aspects of diet quality or a DQS component from the start, hence excluding this poor performing variable will not impact these. We speculate whether the food item category is too broad, and in future versions of MyFoodMonth, we suggest, specifying that tomato should be reported in the vegetable food item, and that the tomato sauce food item should be excluded.

## Conclusions

The relative validity of the non-quantitative 33-item dietary screener ‘MyFoodMonth 1.1’ showed moderate-to-strong concordance and performed satisfactorily in ranking intake for most raw measures, aspects of diet quality, including calcium and iodine, and DQS components compared to a semi-quantitative FFQ, both for men and women in a young student population. This dietary screener presents a promising alternative as a rapid dietary assessment instrument with the potential to reach populations difficult to recruit using traditional instruments.

## Supplementary material

For supplementary material accompanying this paper visit https://doi.org/10.1017/jns.2023.57. click here to view supplementary material
