# Supplementary material for: Relative validity of a non-quantitative 33-item dietary screener with a semi-quantitative food frequency questionnaire among young adults
Source: J Nutr Sci. 2023 Jul 6;12:e72. doi: 10.1017/jns.2023.57 (PMC10345784; doi:10.1017/jns.2023.57)
Supplement: Supplementary file 1 [file jnssup.zip › S2048679023000575sup001.pdf]

Short questionnaire about your diet (MyFoodMonth 1.1)

The questions apply to selected food and beverage items consumed during the last month, that is, the last 30 days. Enter how often you ate or drank the foods and beverages listed below either at meals or as a snack (in-between-meal), at home, on the go, in a café, or anywhere.

Enter (X) to indicate your answer. Thanks for your help!

1. How often did you eat/drink the following items the last month? (Enter <u>once</u> per row)	Times per day				Times per week			Times per month		Never
	6+	4-5	2-3	1	5-6	2-4	1	2-3	1	
**Cereal and porridge**										
Sweetened (e.g., Special K, Corn Flakes with honey)										
Unsweetened (e.g., 4-Korn muesli, oatmeal, Go'dag muesli, and Weetabix)										
**Whole grain bread, crispbread, rolls (>50% whole grain)**										
**Fish spread (e.g., mackerel in tomato sauce)**										
**White cheese (all types)**										
**Whey cheese**										
**Yoghurt, skyr (all types)**										
**Cow's milk (all types)**										
**Plant-based milk (all types)**										
**Juice/smoothie (not nectar)**										
**Fruit and berries, including fresh, frozen, and canned (not juice or smoothie)**										
**Unsalted nuts and seeds**										
**Vegetables, including salad, cabbage, carrot, green beans, etc. (not potatoes or sweet potatoes)**										
**Beans, lentils, chickpeas, peas (not green beans)**										
**Fried potatoes / sweet potatoes (e.g., fries, roast potatoes)**										
**Potatoes / sweet potatoes, other (e.g., baked, boiled, mashed)**										
**Whole grain dinner products (e.g., barley, pasta, couscous)**										
**Pizza (all types)**										
**Tomato sauce, including sauce/salsa for tacos, ketchup, pasta, etc. (not pizza)**										
**Plant-based substitutes (all types of meat substitutes)**										
**Red meat, minced or cuts (beef, lamb/mutton, pork, kid)**										
**Processed meat (e.g., bacon, spread, sausage)**										
**Fatty fish and fish products (e.g., salmon, mackerel)**										
**Lean fish and fish products (e.g., cod, pollock)**										
**Salty snacks (e.g., popcorn, chips, salty nuts)**										
**Candy, including chocolate**										
**Waffles, buns, cake, biscuits etc.**										
**Ice cream, panna cotta, pudding, mousse, etc.**										
**Sugar-sweetened beverages**										
**Sugar-sweetened energy drinks (e.g., Gatorade, Red Bull)**										
**Coffee / tea / iced coffee / iced tea with sugar/syrup/honey**										
**Alcoholic beverages**										
**Water**										

2. Have you taken any supplements such as vitamins, protein supplement etc.?
No ☐<br>Yes ☐
If yes; what and how often?

3. How often do you usually eat the following meals per week? (Enter <u>once</u> per row)								
	Times per week							Rarely/ never
	7	6	5	4	3	2	1	
**Breakfast**								
**Lunch**								
**Dinner**								
**Supper**								
	Times per day				Times per week			Rarely/ never
	6+	4-5	2-3	1	5-6	2-4	1	
**Snack (in-between-meals)**								

4. I avoid consuming certain foods and beverages because of …: (Enter <u>once</u> per row)			
	No, never	Sometimes	Yes, always
… allergies or intolerance(s)			
… my health			
… my religion			
… my weight			
… climate considerations			
… animal welfare			
… veganism			
… other reasons than those above *(describe below)*			
**Describe the foods and beverages you avoid (if applicable):**			
**Other reasons why you avoid consuming certain foods and beverages (if applicable):**			

Comments related to my diet (if applicable):
