# Supplementary material for: Relative validity of a non-quantitative 33-item dietary screener with a semi-quantitative food frequency questionnaire among young adults
Source: J Nutr Sci. 2023 Jul 6;12:e72. doi: 10.1017/jns.2023.57 (PMC10345784; doi:10.1017/jns.2023.57)
Supplement: Supplementary file 1 [file jnssup.zip › S2048679023000575sup002.docx]

| **Diet Quality Score components**  Screener variable(s) | **Scoring Valence** | **Criteria for min score (0)** | **Criteria for max score (10)** | **‘MyFoodMonth 1.1’ Diet Quality Score scorings** | | | | | | | | | |
| --- | --- | --- | --- | --- | --- | --- | --- | --- | --- | --- | --- | --- | --- |
|  |  |  |  | **Never** | **1 a month** | **2-3 a month** | **1 a week** | **2-4 a week** | **5-6 a week** | **1 a day** | **2-3 a day** | **4-5 a day** | **≥6 a day** |
| **Vegetables**  Vegetables, including salad, cabbage, carrot, green beans, etc. (not potatoes or sweet potatoes) | positive | ≤1 x month | ≥4 x day | 0 | 0 | 1 | 2 | 4 | 6 | 8 | 9 | 10 | 10 |
| **Fruits**  Fruit and berries, including fresh, frozen, and canned (not juice or smoothie) | positive | ≤1 x month | ≥2 x day | 0 | 0 | 1 | 2 | 4 | 6 | 8 | 10 | 10 | 10 |
| **Whole grain (products)**  Cereal and porridge, Unsweetened (e.g., 4-Korn muesli, oatmeal, Go’dag muesli, and Weetabix)  Whole grain bread, crispbread, rolls (>50% whole grain)  Whole grain dinner products (e.g., barley, pasta, couscous) | positive | ≤1 x month | 2-5 x day | 0 | 0 | 1 | 2 | 4 | 6 | 8 | 10 | 10 | 8 |
| **Sugar-sweetened beverages**  Sugar-sweetened beverages  Sugar-sweetened energy drinks (e.g., Gatorade, Red Bull) | negative | ≥1 x day | 0 | 10 | 9 | 8 | 6 | 4 | 1 | 0 | 0 | 0 | 0 |
| **Sugary foods**  Cereal and porridge, Sweetened (e.g., Special K, Corn Flakes with honey)  Candy, including chocolate  Waffles, buns, cake, biscuits etc.  Ice cream, panna cotta, pudding, mousse, etc. | negative | ≥1 x day | 0 | 10 | 9 | 8 | 6 | 4 | 1 | 0 | 0 | 0 | 0 |
| **Beans and lentils**  Beans, lentils, chickpeas, peas (not green beans) | positive | 0 | ≥2 x day | 0 | 1 | 2 | 4 | 6 | 8 | 9 | 10 | 10 | 10 |
| **Nuts and seeds (unsalted)**  Unsalted nuts and seeds | positive | 0 | 1-3 x day | 0 | 1 | 2 | 4 | 6 | 8 | 10 | 10 | 8 | 6 |
| **Meat (processed and red)**  Red meat, minced or cuts (beef, lamb, pork, goat)  Processed meat (e.g., bacon, spread, sausage) | negative | ≥2 x day | ≤1 x month | 10 | 10 | 8 | 6 | 4 | 2 | 1 | 0 | 0 | 0 |
| **Fish**  Fatty fish and fish products (e.g., salmon, mackerel)  Lean fish and fish products (e.g., cod, pollock)  Fish spread (e.g., mackerel in tomato sauce) | Positive | 0 | ≥1 x week | 0 | 4 | 7 | 10 | 10 | 10 | 10 | 10 | 10 | 10 |
| **Salty foods**  Salty snacks (e.g., popcorn, chips, salty nuts) | negative | ≥2 x day | 0 | 10 | 9 | 8 | 6 | 4 | 2 | 1 | 0 | 0 | 0 |
|  |  |  |  |  |  |  |  |  | **Total Possible Points:** | | | | **100** |

**Supplementary file 3. ’MyFoodMonth 1.1’ Diet Quality Score scorings**
