# Supplementary material for: Relative validity of a non-quantitative 33-item dietary screener with a semi-quantitative food frequency questionnaire among young adults
Source: J Nutr Sci. 2023 Jul 6;12:e72. doi: 10.1017/jns.2023.57 (PMC10345784; doi:10.1017/jns.2023.57)

Supplementary file 4.

Figure S1-S31. Box and whisker plots of 31 items in the dietary screener 'MyFoodMonth 1.1' compared to a semi-quantitative food-frequency questionnaire. Participants presented pooled, the box indicating median and interquartile range (25th and 75th percentiles), and whiskers as quartile ±1.5*interquartile range convention.

Figure S1. Box and whisker plot for “Cereal & porridge (sweetened)” in the dietary screener 'MyFoodMonth 1.1' compared to a semi-quantitative food-frequency questionnaire. Participants presented pooled, the box indicating median and interquartile range (25th and 75th percentiles), and whiskers as quartile ±1.5*interquartile range convention.

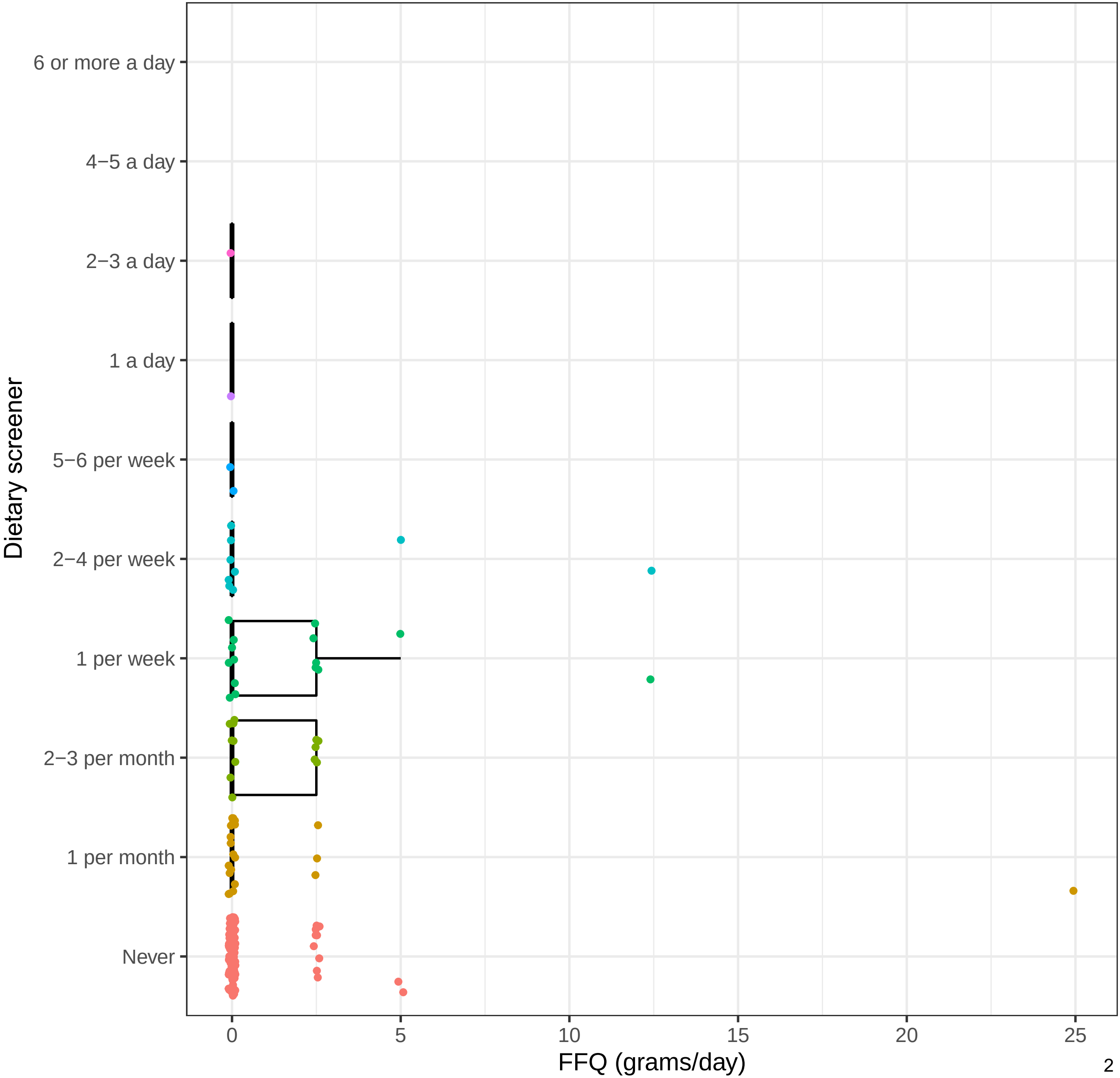

Figure S2. Box and whisker plot for “Cereal & porridge (unsweetened)” in the dietary screener 'MyFoodMonth 1.1' compared to a semi-quantitative food-frequency questionnaire. Participants presented pooled, the box indicating median and interquartile range (25th and 75th percentiles), and whiskers as quartile ±1.5*interquartile range convention.

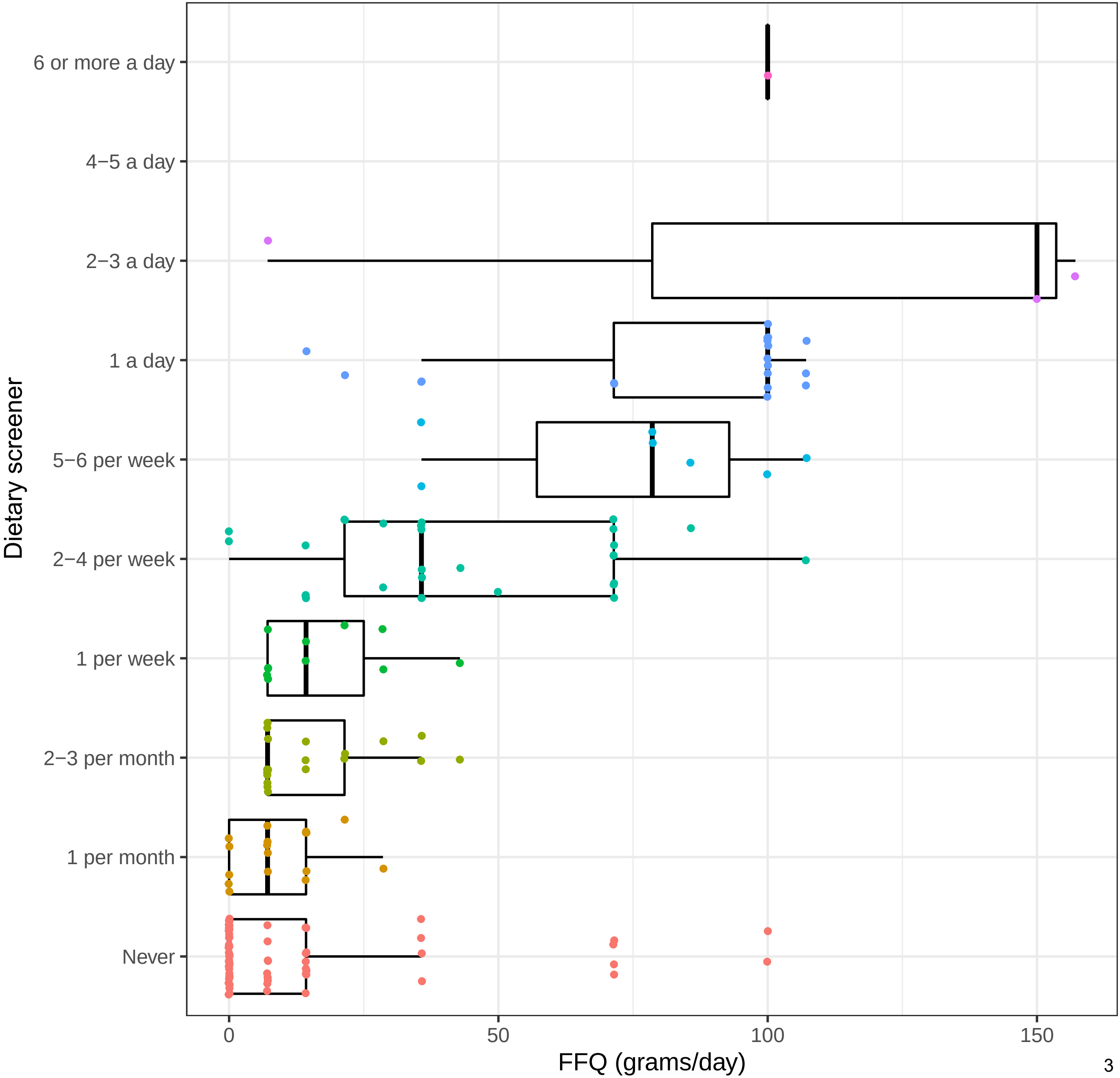

Figure S3. Box and whisker plot for “Whole−grain bread” in the dietary screener 'MyFoodMonth 1.1' compared to a semi-quantitative food-frequency questionnaire. Participants presented pooled, the box indicating median and interquartile range (25th and 75th percentiles), and whiskers as quartile ±1.5*interquartile range convention.

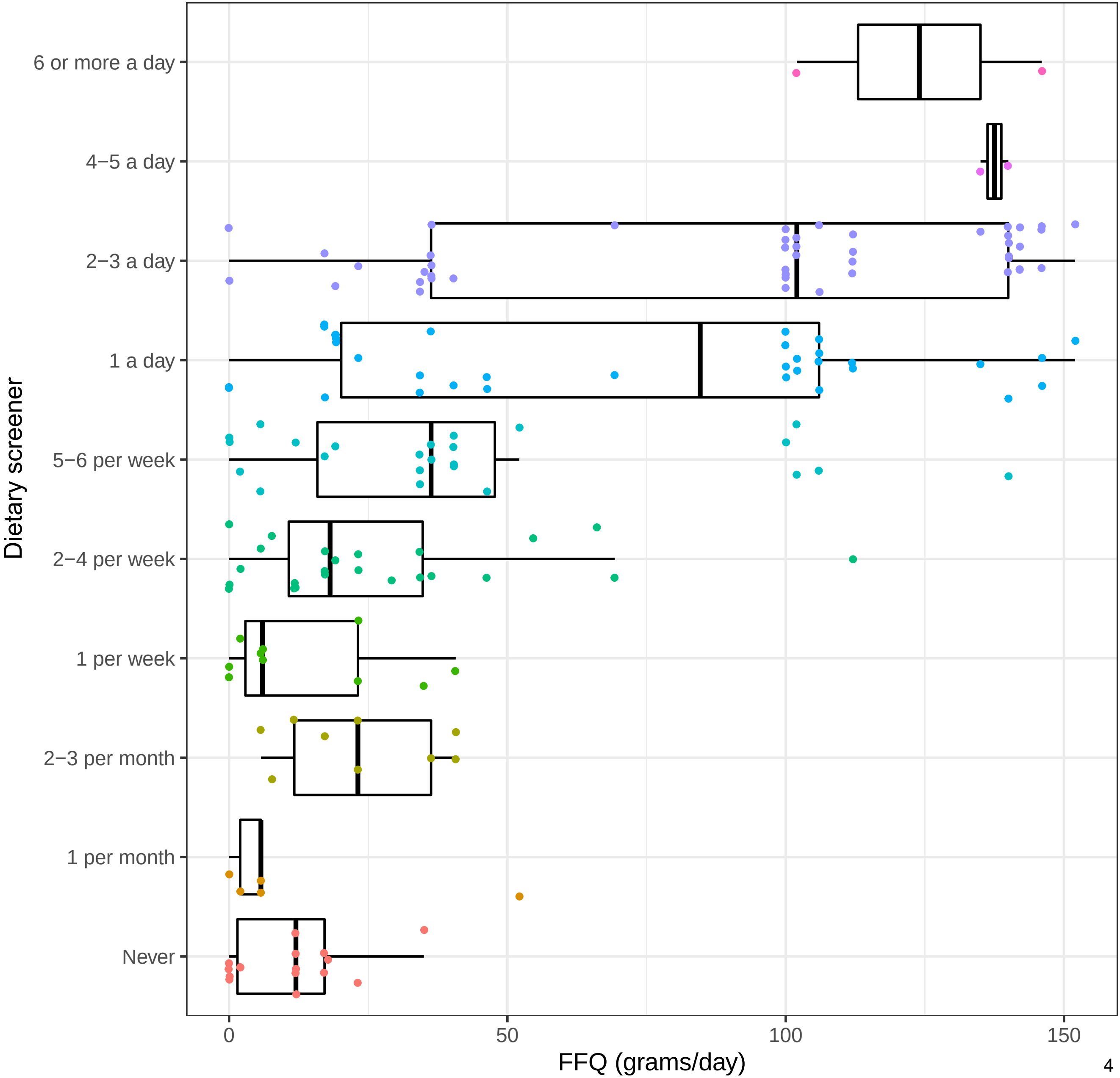

Figure S4. Box and whisker plot for “Fish spread” in the dietary screener 'MyFoodMonth 1.1' compared to a semi-quantitative food-frequency questionnaire. Participants presented pooled, the box indicating median and interquartile range (25th and 75th percentiles), and whiskers as quartile ±1.5*interquartile range convention.

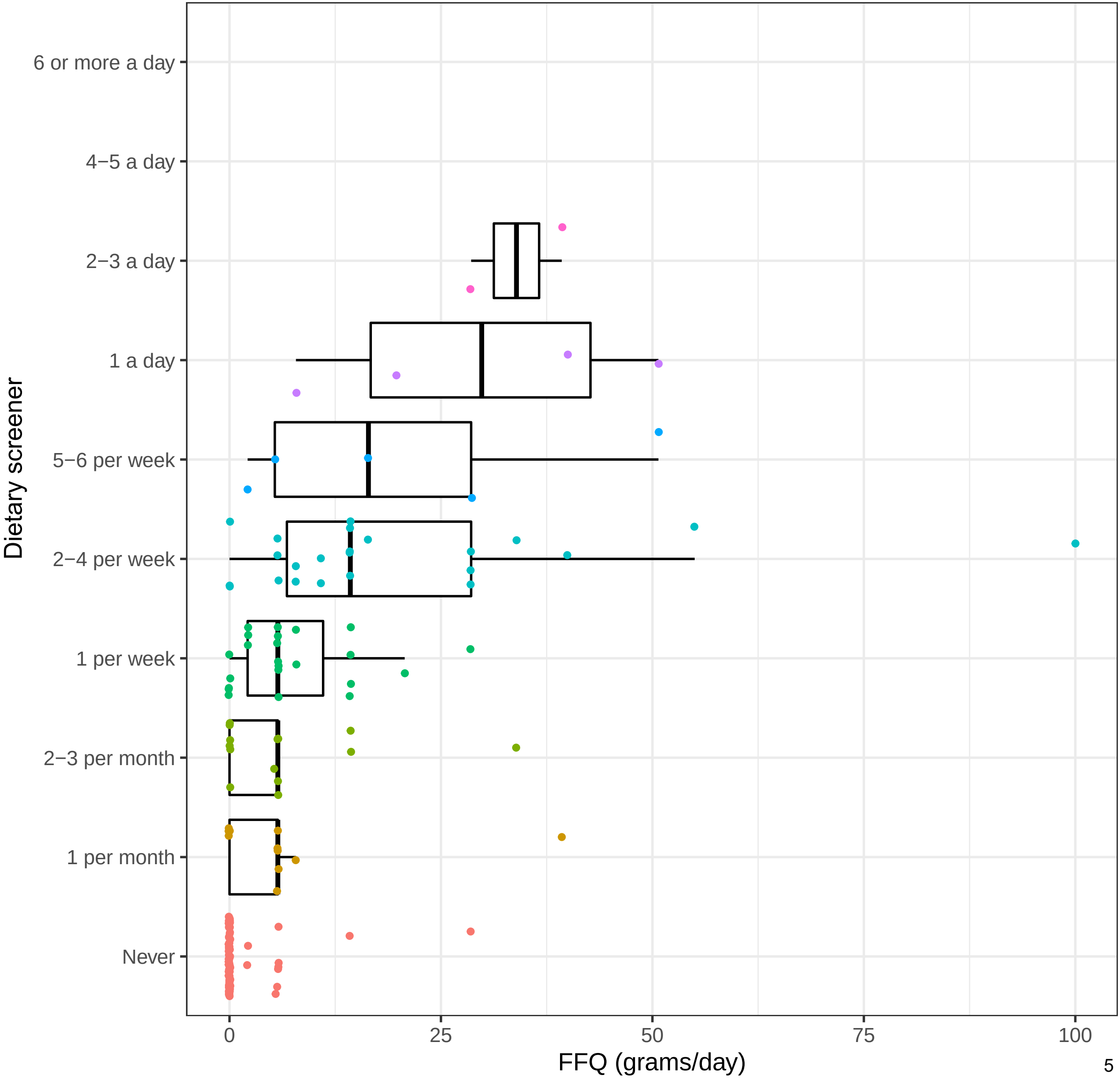

Figure S5. Box and whisker plot for “White cheese” in the dietary screener 'MyFoodMonth 1.1' compared to a semi-quantitative food-frequency questionnaire. Participants presented pooled, the box indicating median and interquartile range (25th and 75th percentiles), and whiskers as quartile 1.5*interquartile range convention.

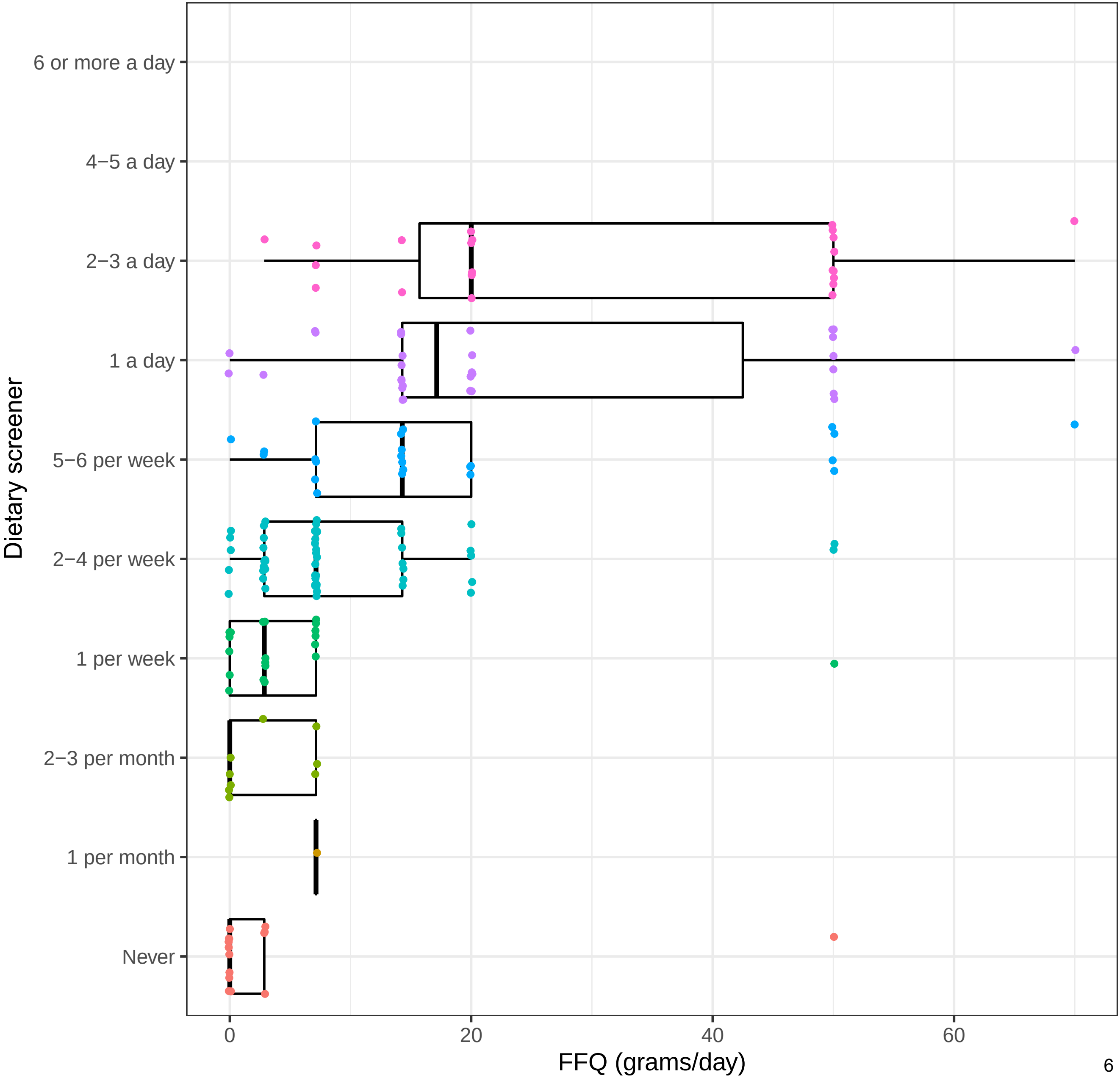

Figure S6. Box and whisker plot for “Whey cheese” in the dietary screener 'MyFoodMonth 1.1' compared to a semi-quantitative food-frequency questionnaire. Participants presented pooled, the box indicating median and interquartile range (25th and 75th percentiles), and whiskers as quartile ±1.5*interquartile range convention.

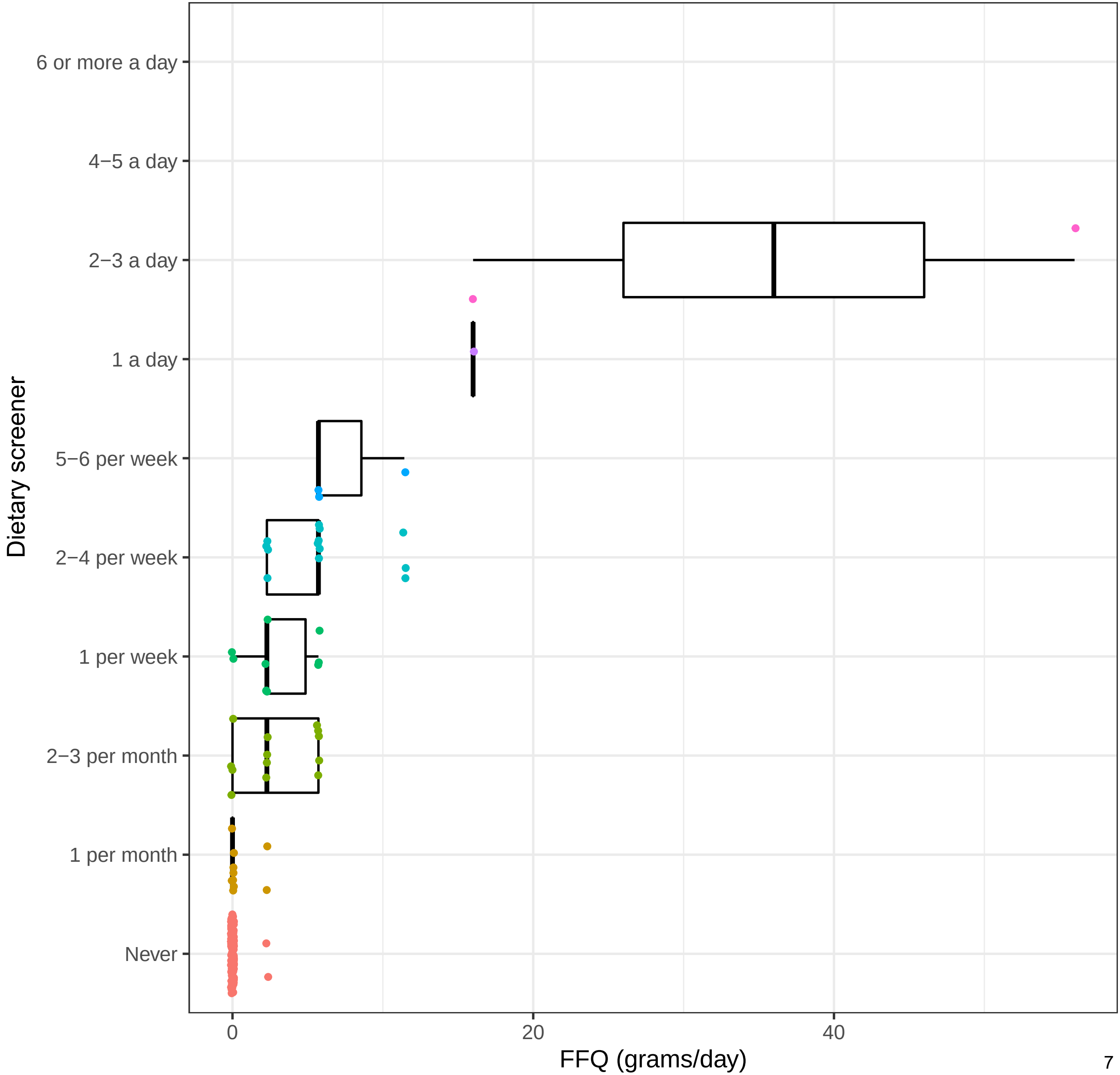

Figure S7. Box and whisker plot for “Yoghurt” in the dietary screener 'MyFoodMonth 1.1' compared to a semi-quantitative food-frequency questionnaire. Participants presented pooled, the box indicating median and interquartile range (25th and 75th percentiles), and whiskers as quartile ±1.5*interquartile range convention.

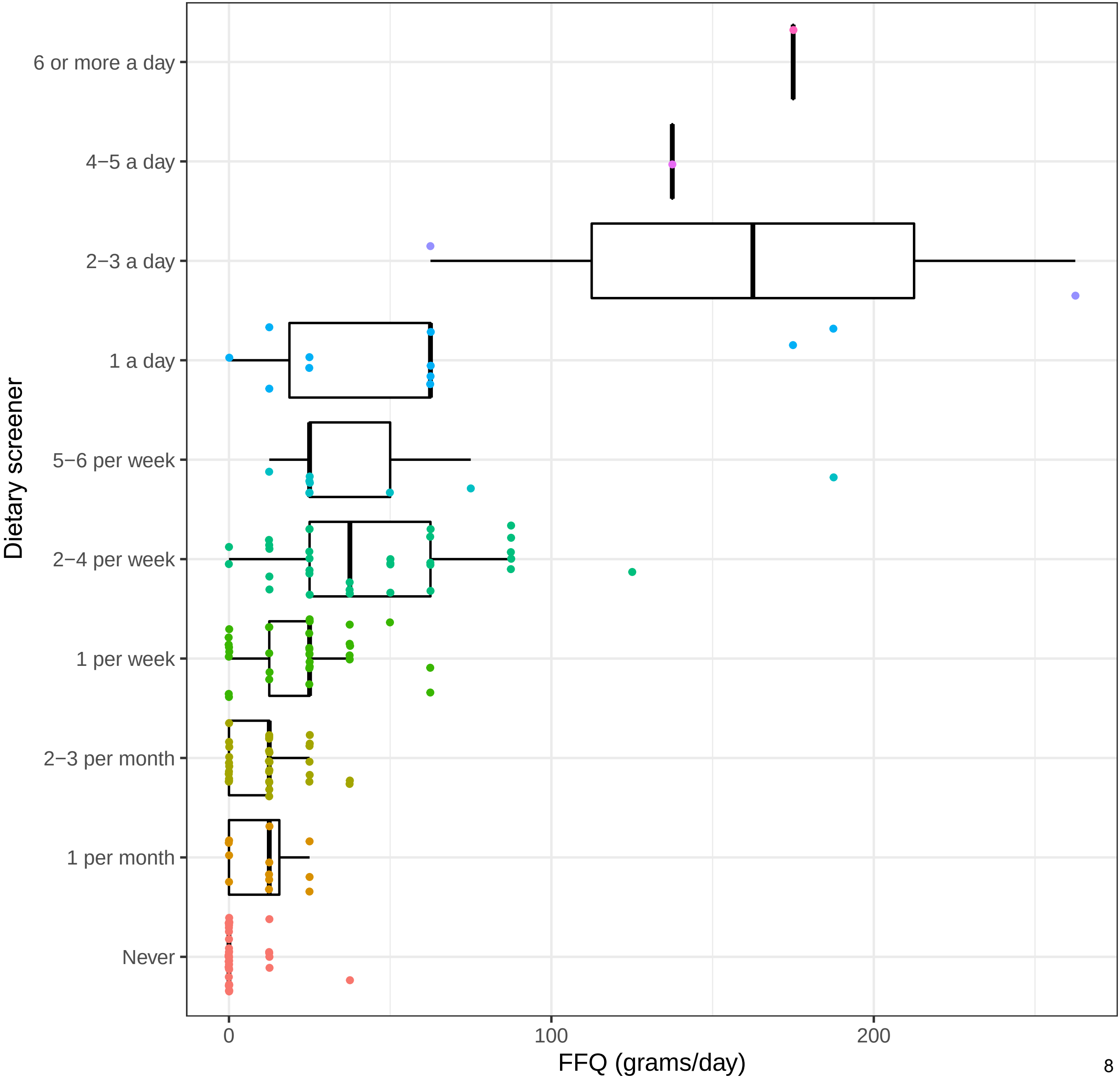

Figure S8. Box and whisker plot for "Cow milk" in the dietary screener 'MyFoodMonth 1.1' compared to a semi-quantitative food-frequency questionnaire. Participants presented pooled, the box indicating median and interquartile range (25th and 75th percentiles), and whiskers as quartile ±1.5*interquartile range convention.

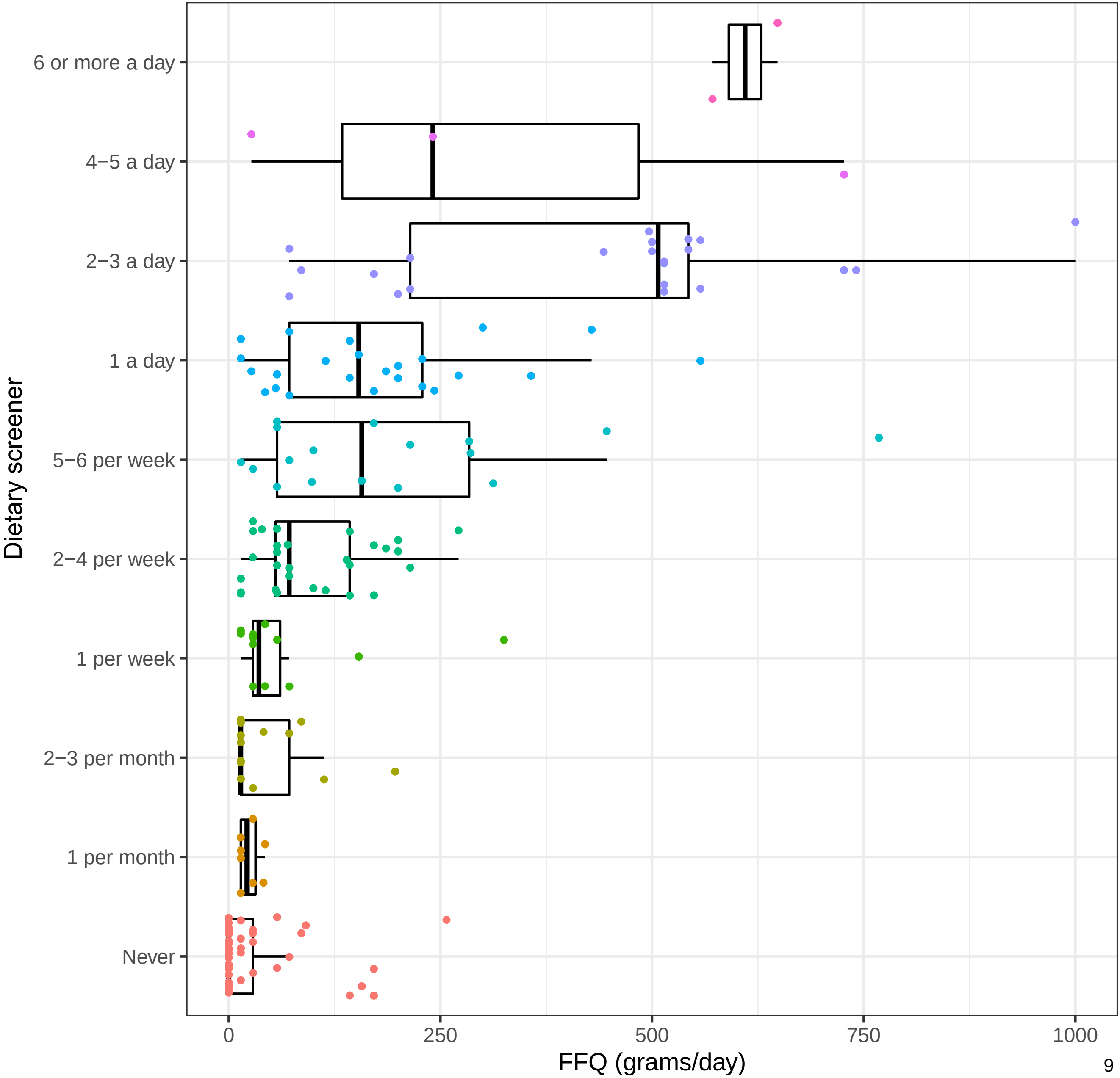

Figure S9. Box and whisker plot for “Plant−based milk” in the dietary screener 'MyFoodMonth 1.1' compared to a semi-quantitative food-frequency questionnaire. Participants presented pooled, the box indicating median and interquartile range (25th and 75th percentiles), and whiskers as quartile ±1.5*interquartile range convention.

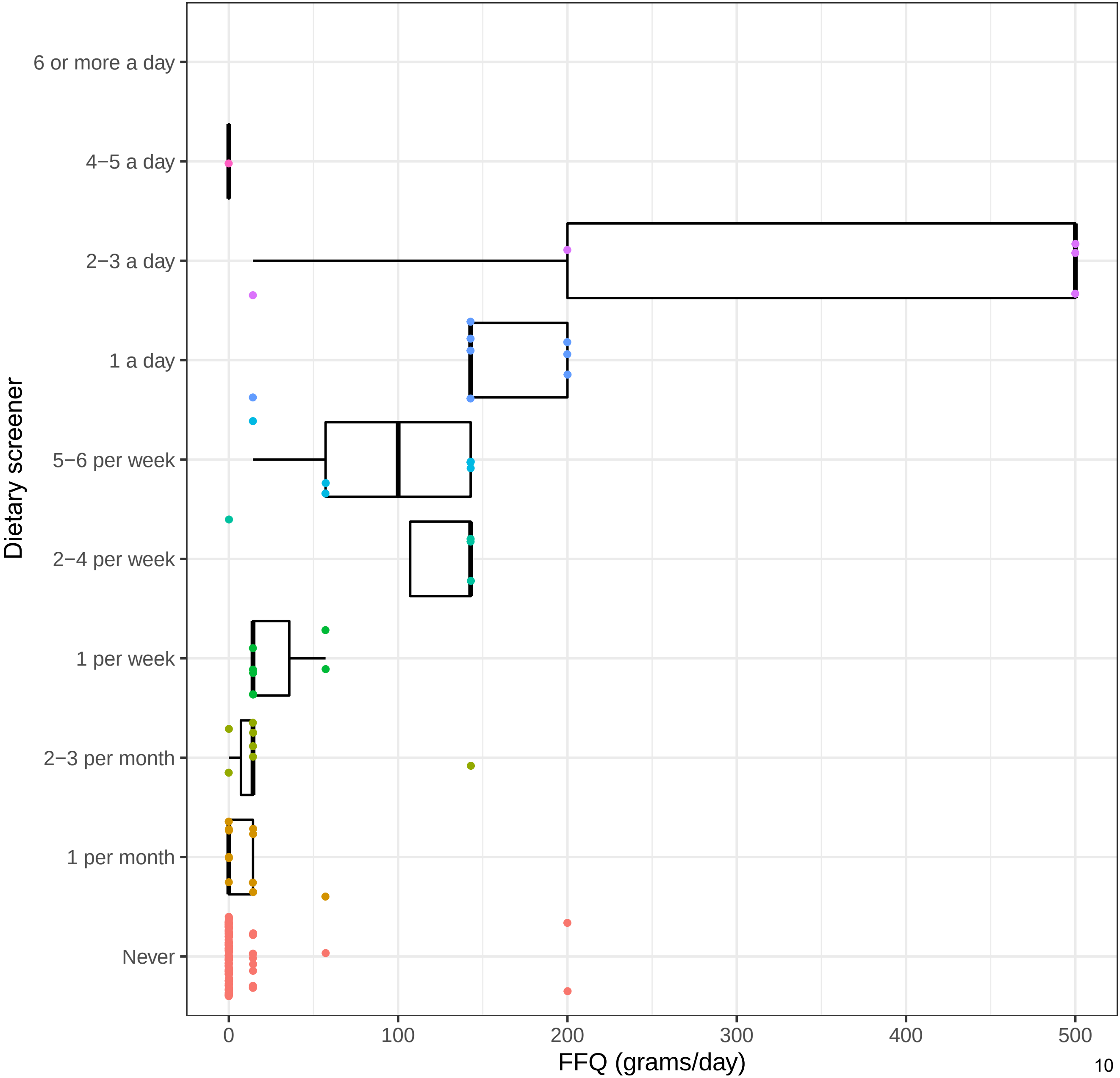

Figure S10. Box and whisker plot for “Juice/Smoothie” in the dietary screener 'MyFoodMonth 1.1' compared to a semi-quantitative food-frequency questionnaire. Participants presented pooled, the box indicating median and interquartile range (25th and 75th percentiles), and whiskers as quartile ±1.5*interquartile range convention.

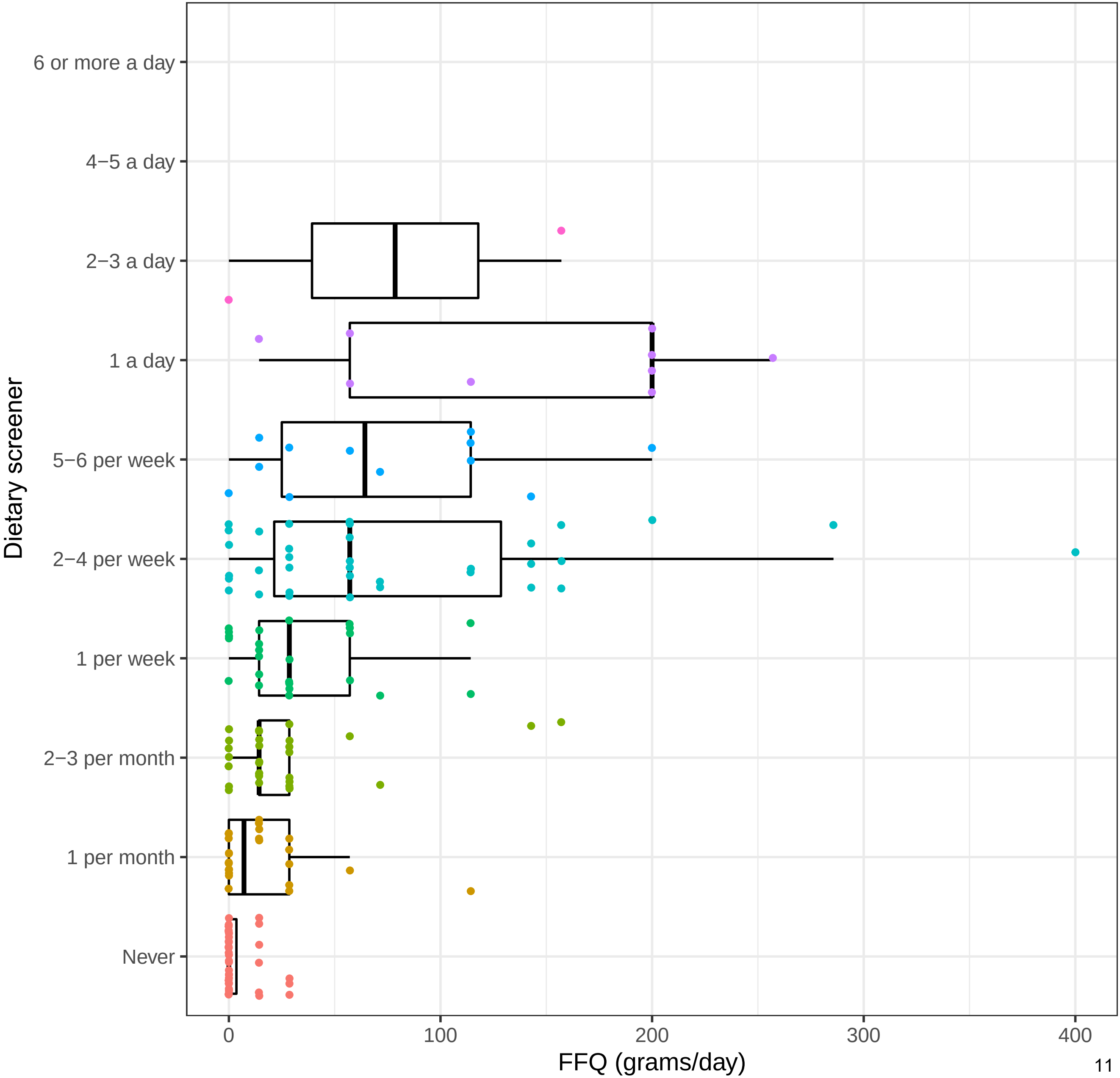

Figure S11. Box and whisker plot for “Fruit/berries (not juice/smoothie)” in the dietary screener 'MyFoodMonth 1.1' compared to a semi-quantitative food-frequency questionnaire. Participants presented pooled, the box indicating median and interquartile range (25th and 75th percentiles), and whiskers as quartile ±1.5*interquartile range convention.

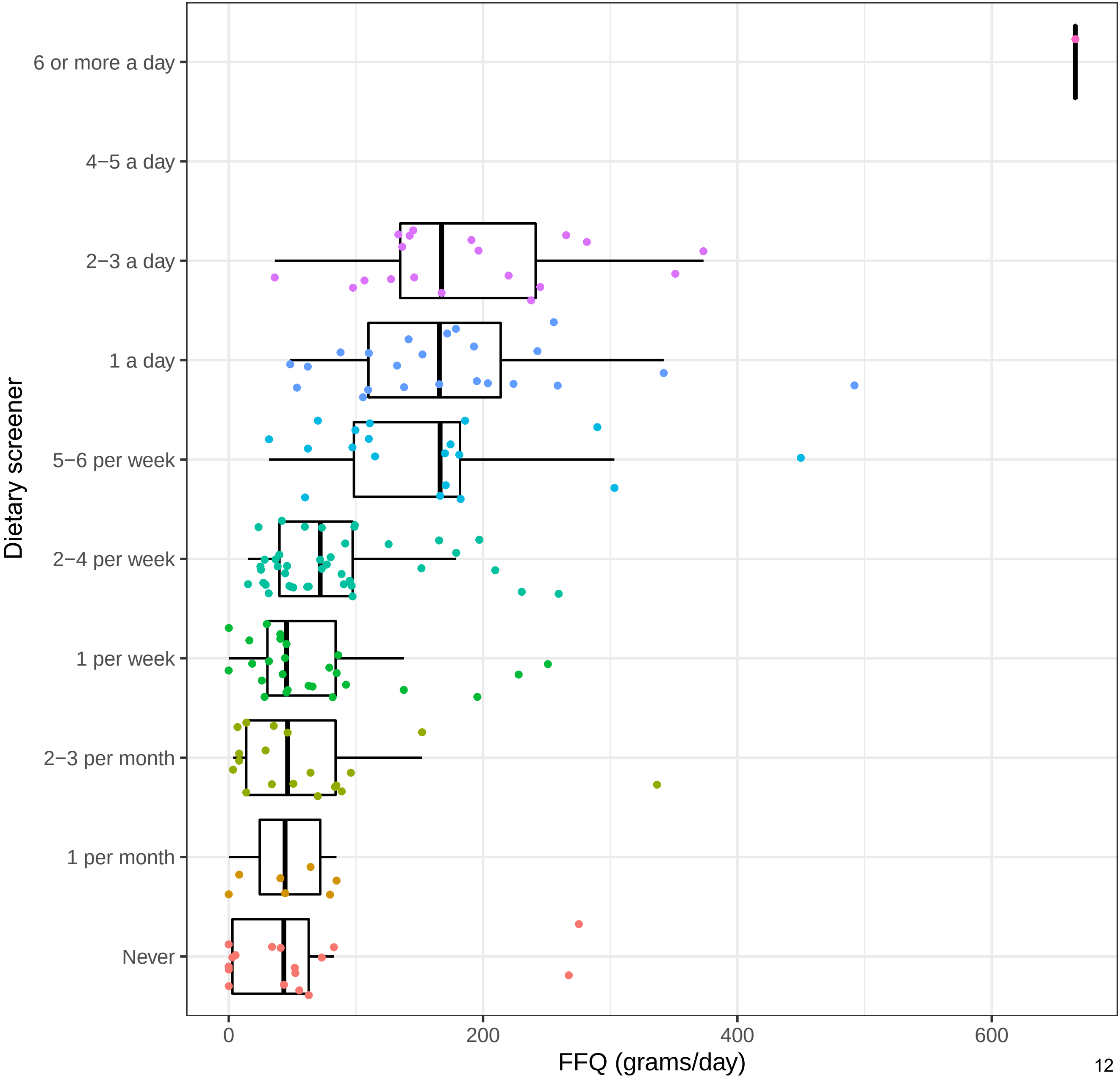

Figure S12. Box and whisker plot for “Vegetables (not potatoes)” in the dietary screener 'MyFoodMonth 1.1' compared to a semi-quantitative food-frequency questionnaire. Participants presented pooled, the box indicating median and interquartile range (25th and 75th percentiles), and whiskers as quartile ±1.5*interquartile range convention.

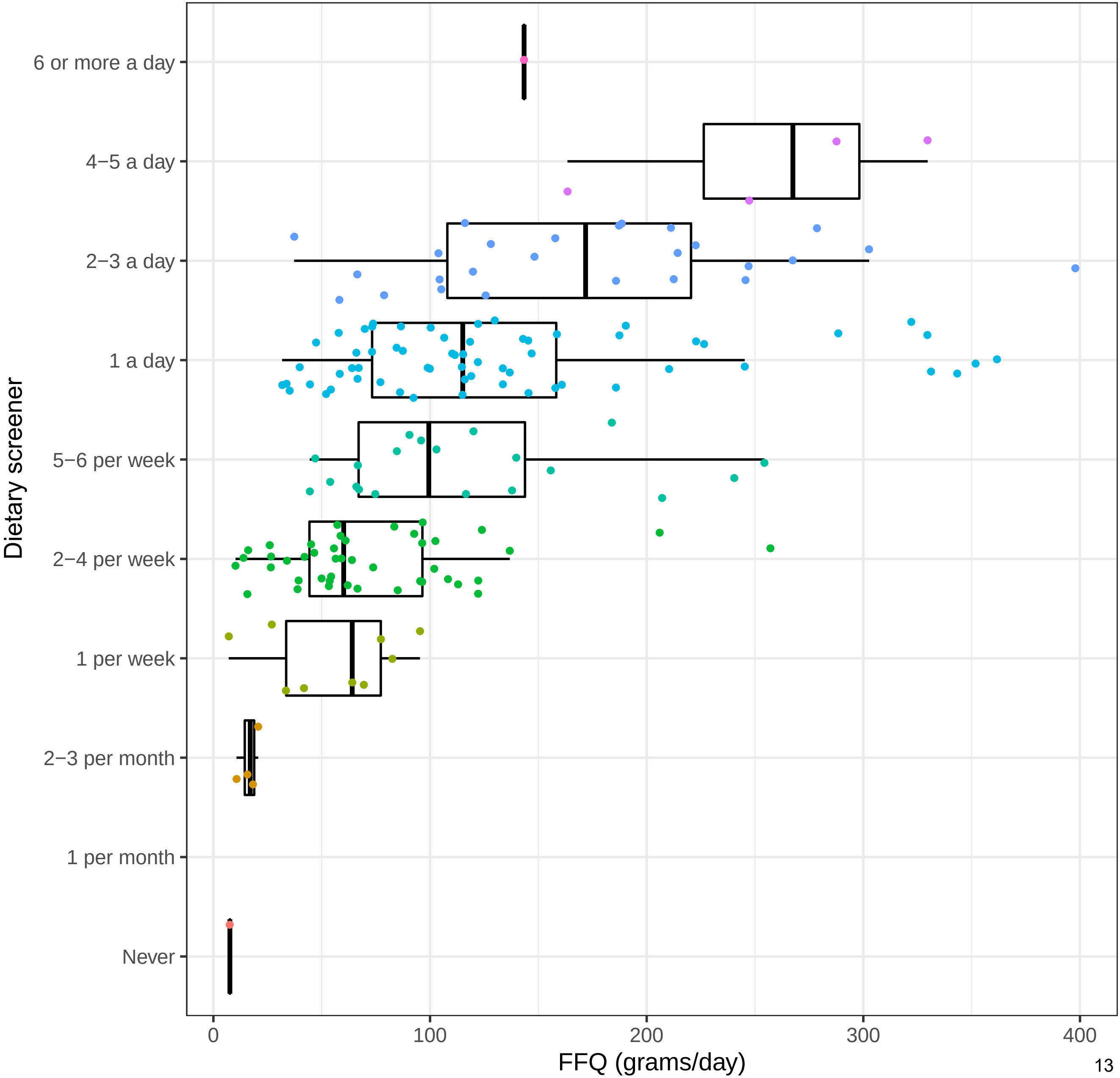

Figure S13. Box and whisker plot for “Beans/lentils/chickpeas/peas” in the dietary screener 'MyFoodMonth 1.1' compared to a semi-quantitative food-frequency questionnaire. Participants presented pooled, the box indicating median and interquartile range (25th and 75th percentiles), and whiskers as quartile ±1.5*interquartile range convention.

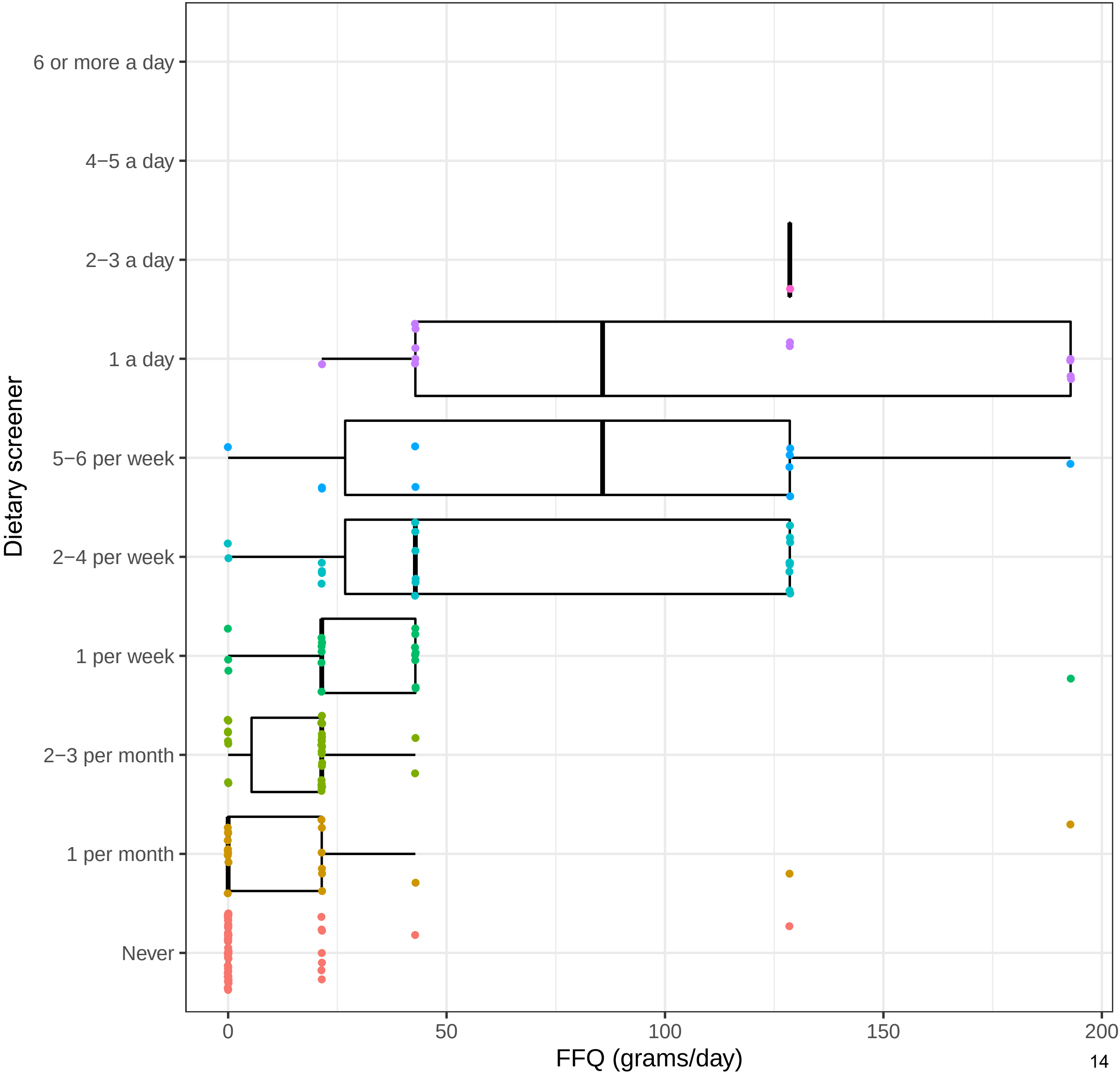

Figure S14. Box and whisker plot for “Fried potatoes/sweet potatoes” in the dietary screener 'MyFoodMonth 1.1' compared to a semi-quantitative food-frequency questionnaire. Participants presented pooled, the box indicating median and interquartile range (25th and 75th percentiles), and whiskers as quartile ±1.5*interquartile range convention.

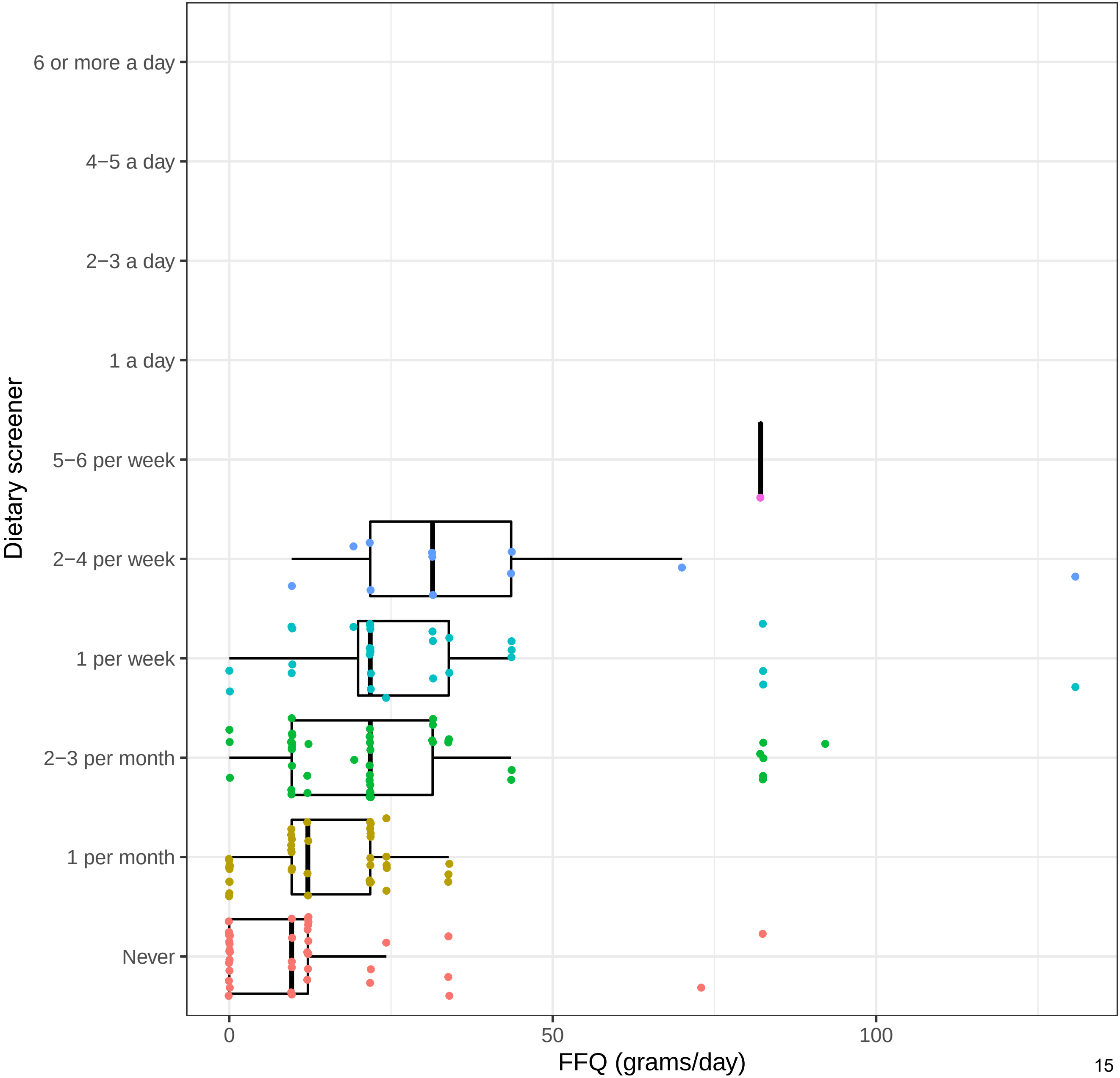

Figure S15. Box and whisker plot for "Potatoes/sweet potatoes" in the dietary screener 'MyFoodMonth 1.1' compared to a semi-quantitative food-frequency questionnaire. Participants presented pooled, the box indicating median and interquartile range (25th and 75th percentiles), and whiskers as quartile ±1.5*interquartile range convention.

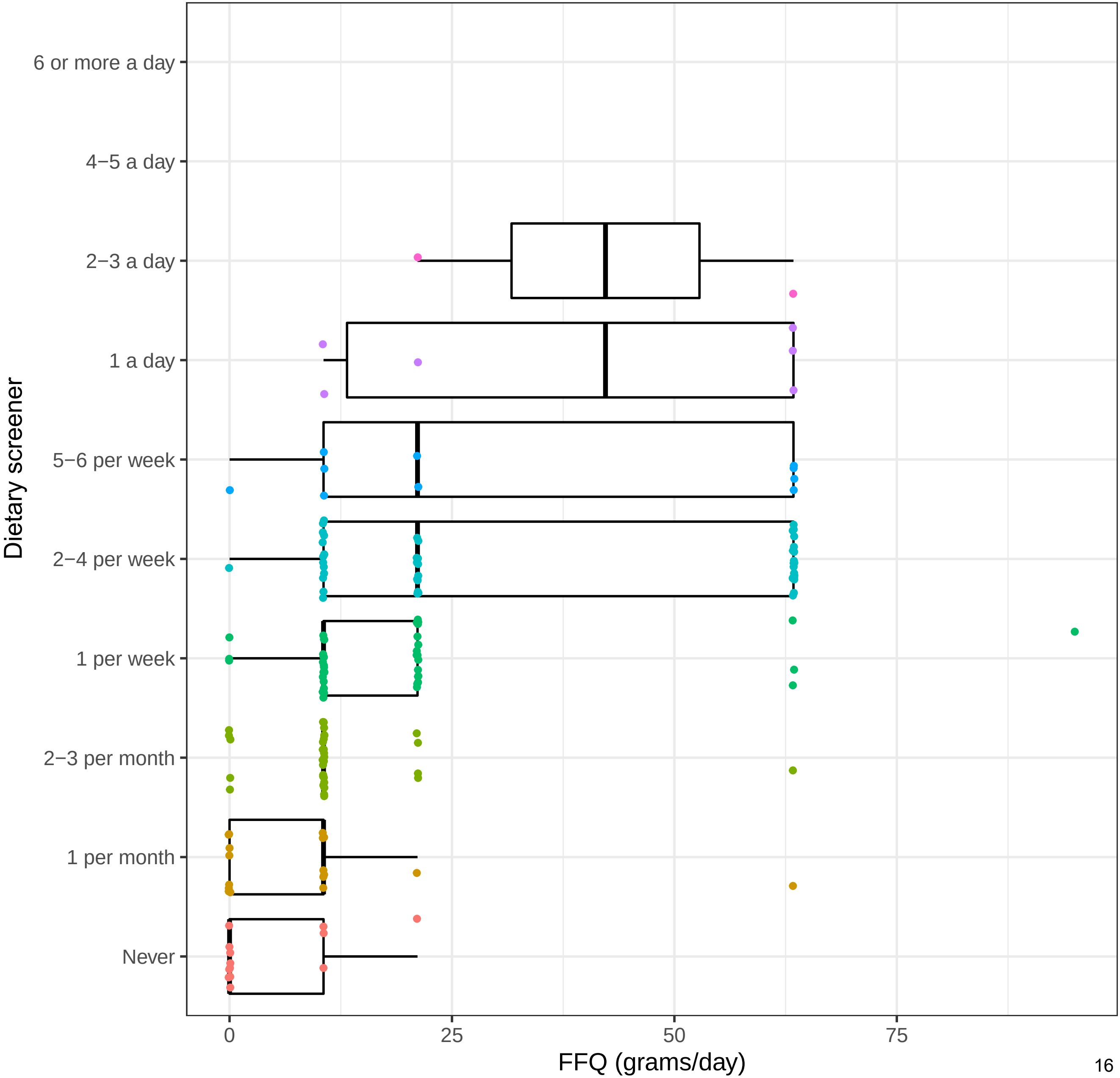

Figure S16. Box and whisker plot for “Whole grain (barley/pasta/couscous)” in the dietary screener 'MyFoodMonth 1.1' compared to a semi-quantitative food-frequency questionnaire. Participants presented pooled, the box indicating median and interquartile range (25th and 75th percentiles), and whiskers as quartile ±1.5*interquartile range convention.

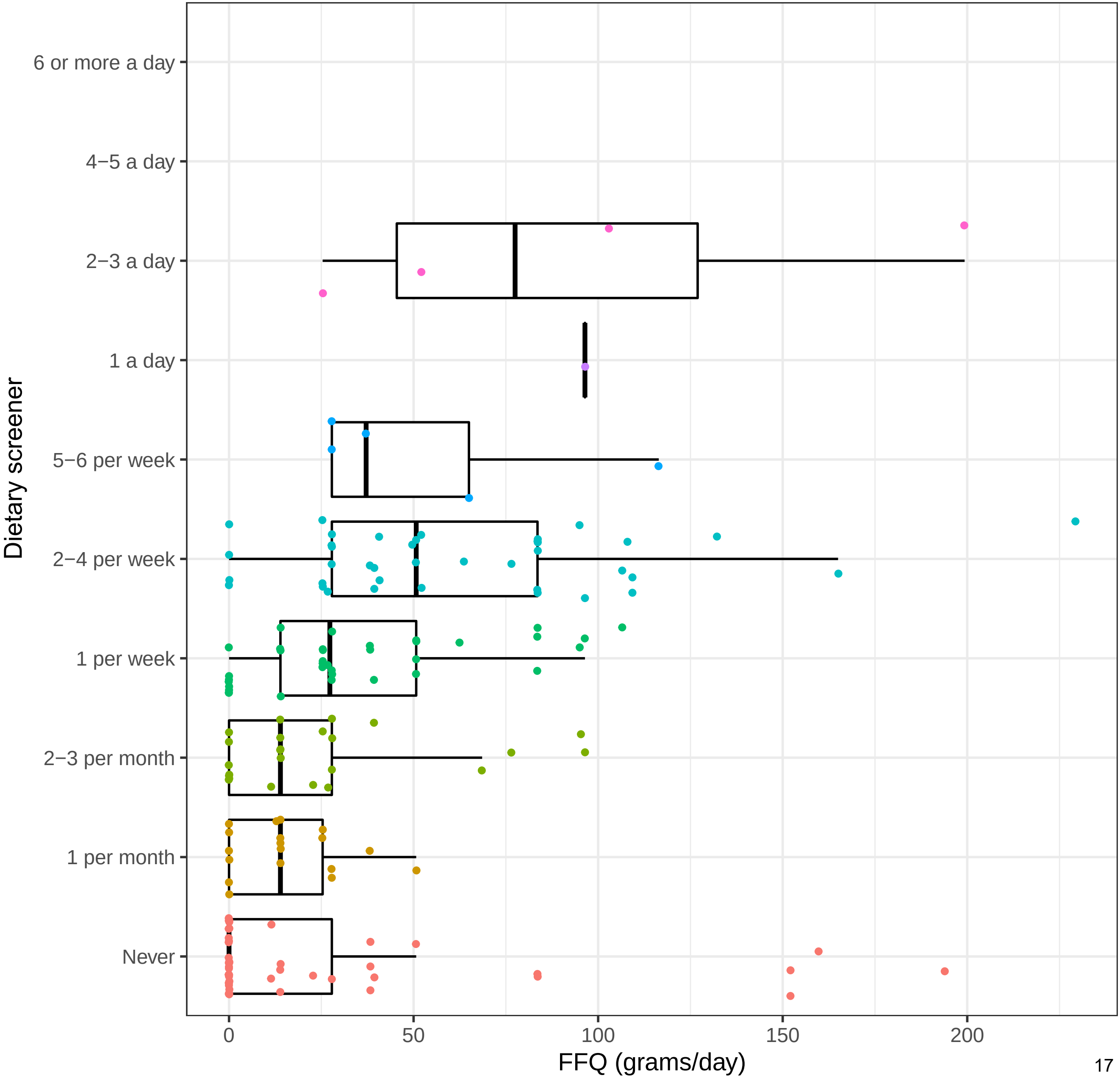

Figure S17. Box and whisker plot for “Pizza” in the dietary screener 'MyFoodMonth 1.1' compared to a semi-quantitative food-frequency questionnaire. Participants presented pooled, the box indicating median and interquartile range (25th and 75th percentiles), and whiskers as quartile ±1.5*interquartile range convention.

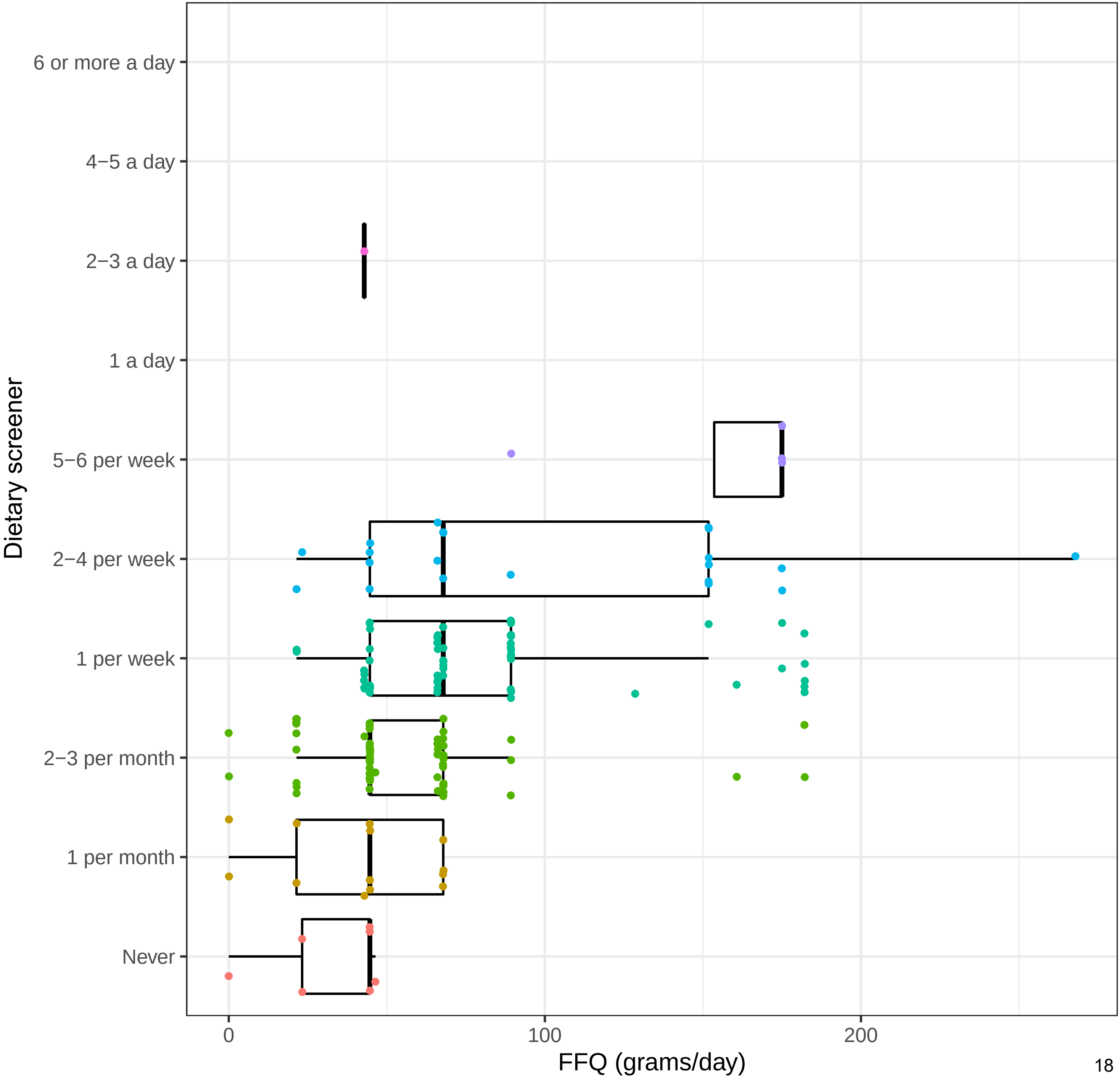

Figure S18. Box and whisker plot for “Tomato sauce” in the dietary screener 'MyFoodMonth 1.1' compared to a semi-quantitative food-frequency questionnaire. Participants presented pooled, the box indicating median and interquartile range (25th and 75th percentiles), and whiskers as quartile ±1.5*interquartile range convention.

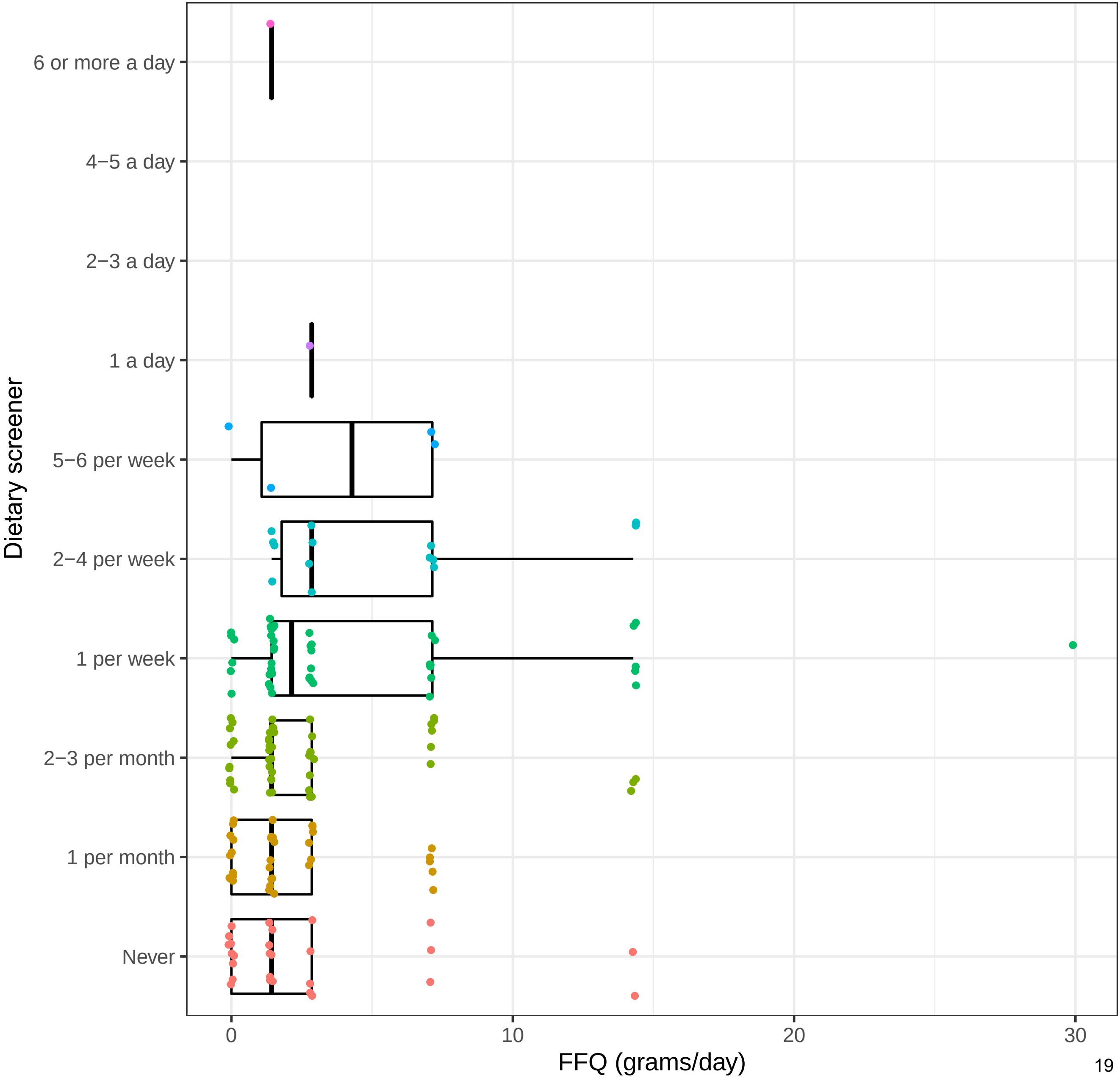

Figure S19. Box and whisker plot for “Red meat (beef/lamb/pork/goat)” in the dietary screener 'MyFoodMonth 1.1' compared to a semi-quantitative food-frequency questionnaire. Participants presented pooled, the box indicating median and interquartile range (25th and 75th percentiles), and whiskers as quartile ±1.5*interquartile range convention.

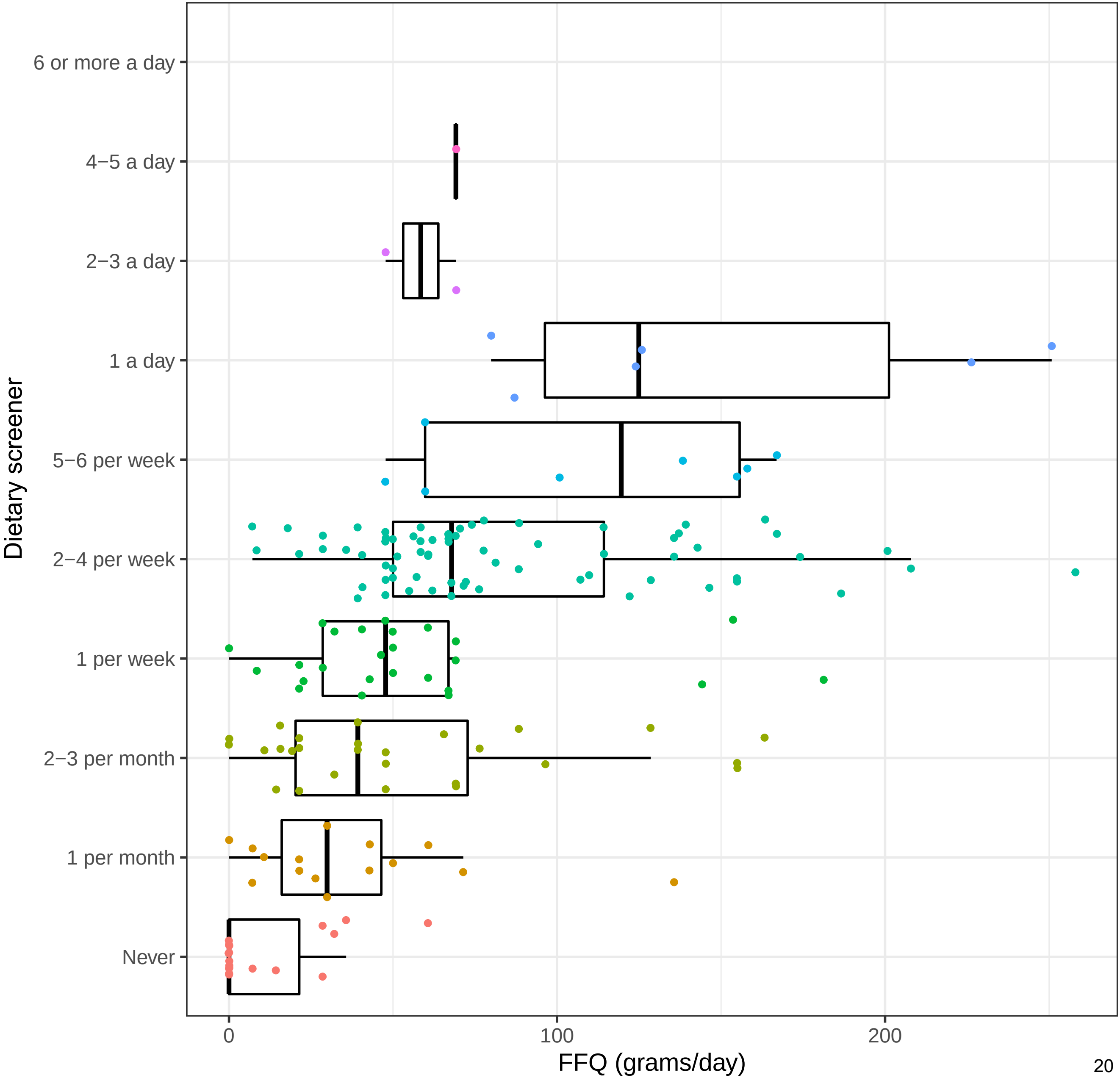

Figure S20. Box and whisker plot for “Processed meat” in the dietary screener 'MyFoodMonth 1.1' compared to a semi-quantitative food-frequency questionnaire. Participants presented pooled, the box indicating median and interquartile range (25th and 75th percentiles), and whiskers as quartile ±1.5*interquartile range convention.

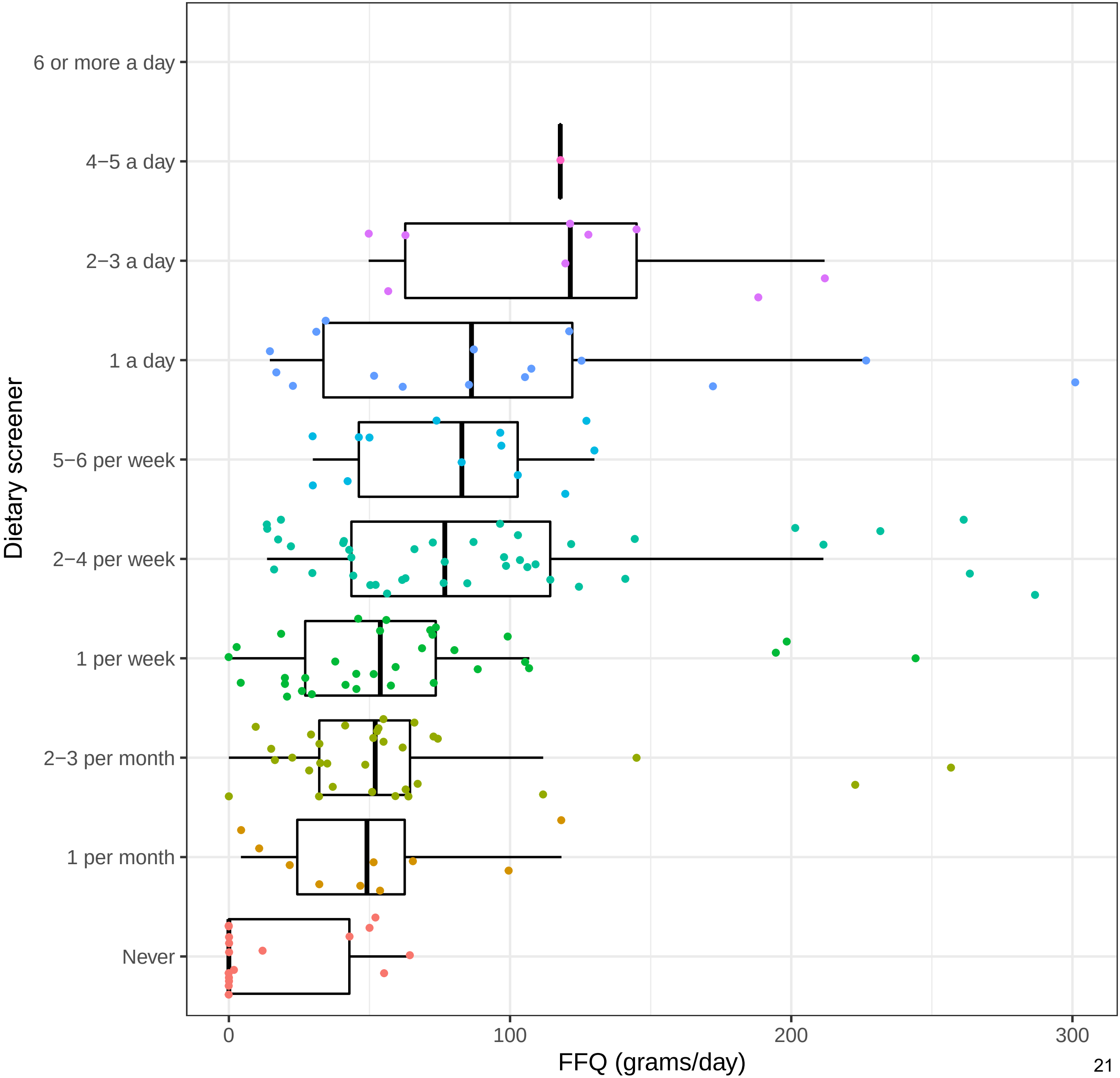

Figure S21. Box and whisker plot for “Fatty fish (salmon/mackerel)” in the dietary screener 'MyFoodMonth 1.1' compared to a semi-quantitative food-frequency questionnaire. Participants presented pooled, the box indicating median and interquartile range (25th and 75th percentiles), and whiskers as quartile ±1.5*interquartile range convention.

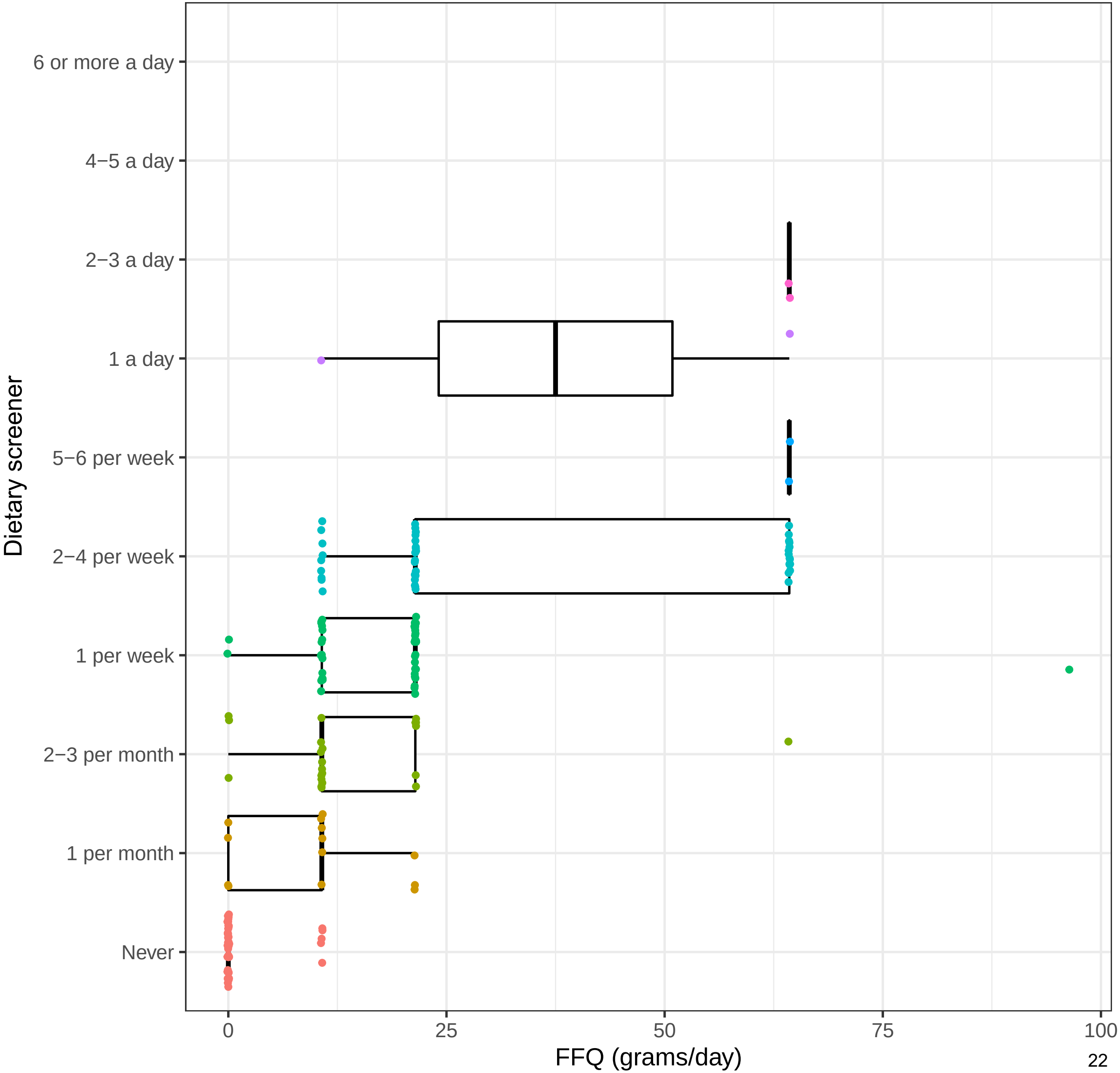

Figure S22. Box and whisker plot for "Lean fish (cod/pollock)" in the dietary screener 'MyFoodMonth 1.1' compared to a semi-quantitative food-frequency questionnaire. Participants presented pooled, the box indicating median and interquartile range (25th and 75th percentiles), and whiskers as quartile ±1.5*interquartile range convention.

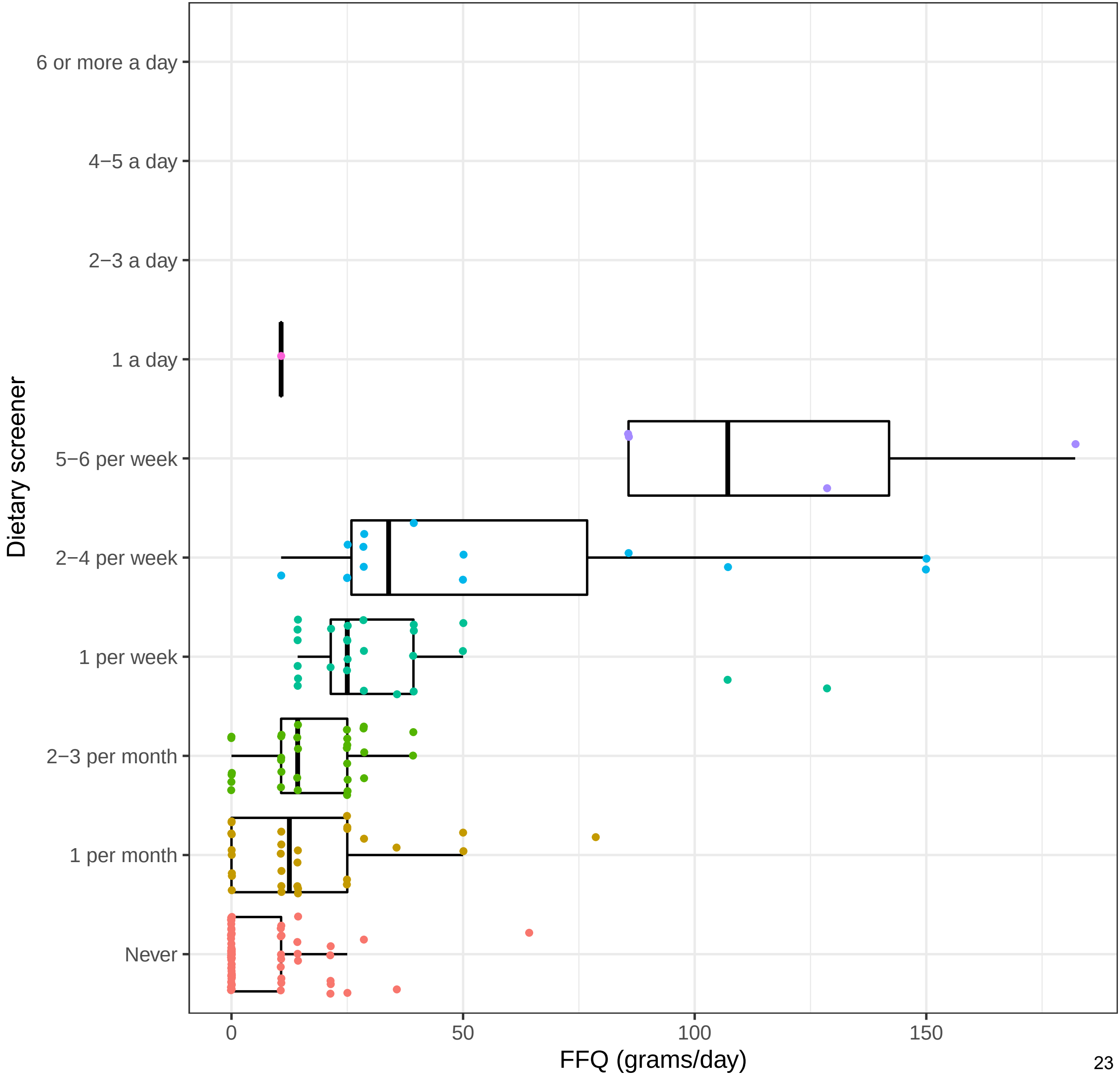

Figure S23. Box and whisker plot for “Salty snacks” in the dietary screener 'MyFoodMonth 1.1' compared to a semi-quantitative food-frequency questionnaire. Participants presented pooled, the box indicating median and interquartile range (25th and 75th percentiles), and whiskers as quartile ±1.5*interquartile range convention.

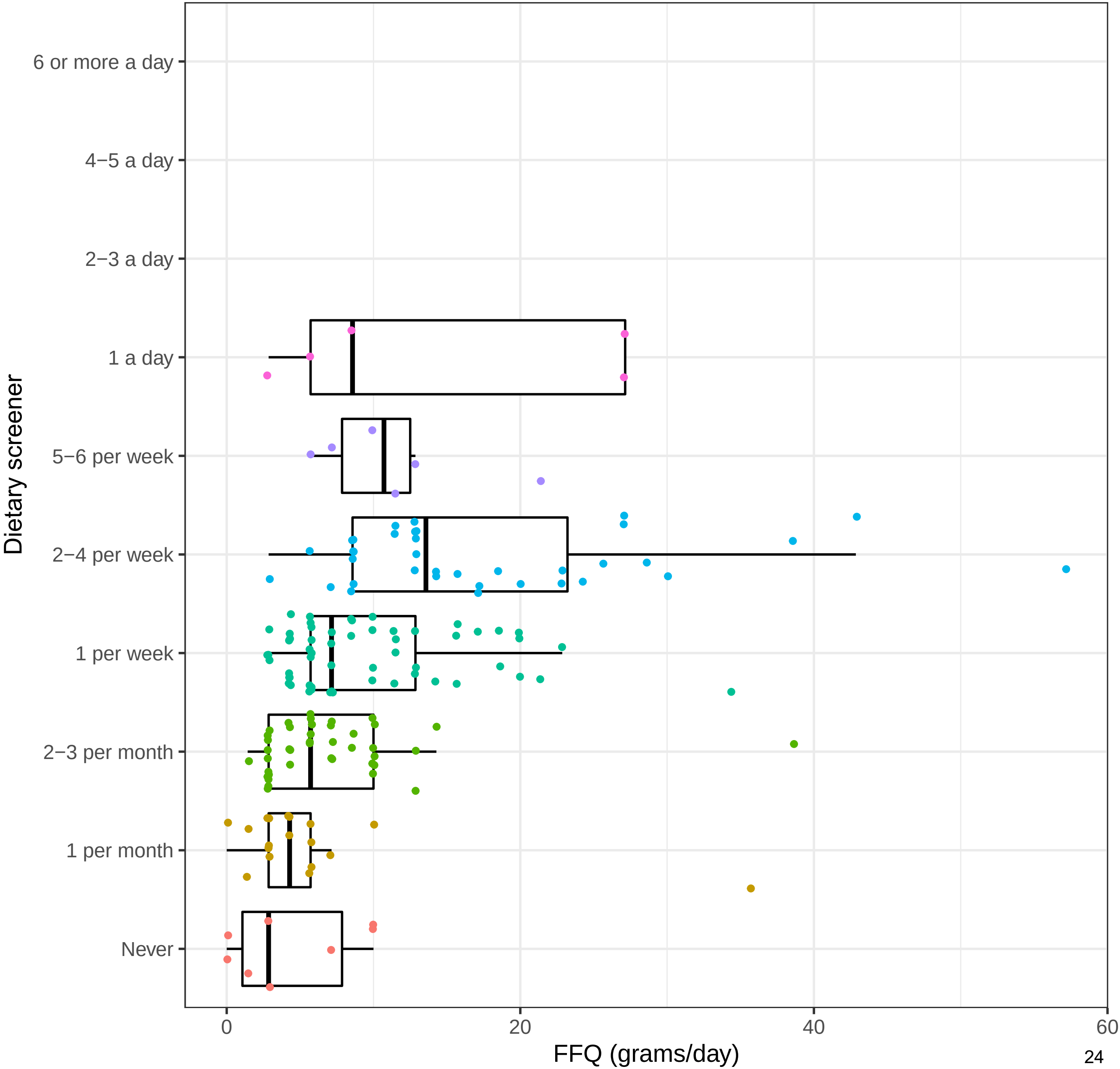

Figure S24. Box and whisker plot for "Candy/chocolate" in the dietary screener 'MyFoodMonth 1.1' compared to a semi-quantitative food-frequency questionnaire. Participants presented pooled, the box indicating median and interquartile range (25th and 75th percentiles), and whiskers as quartile ±1.5*interquartile range convention.

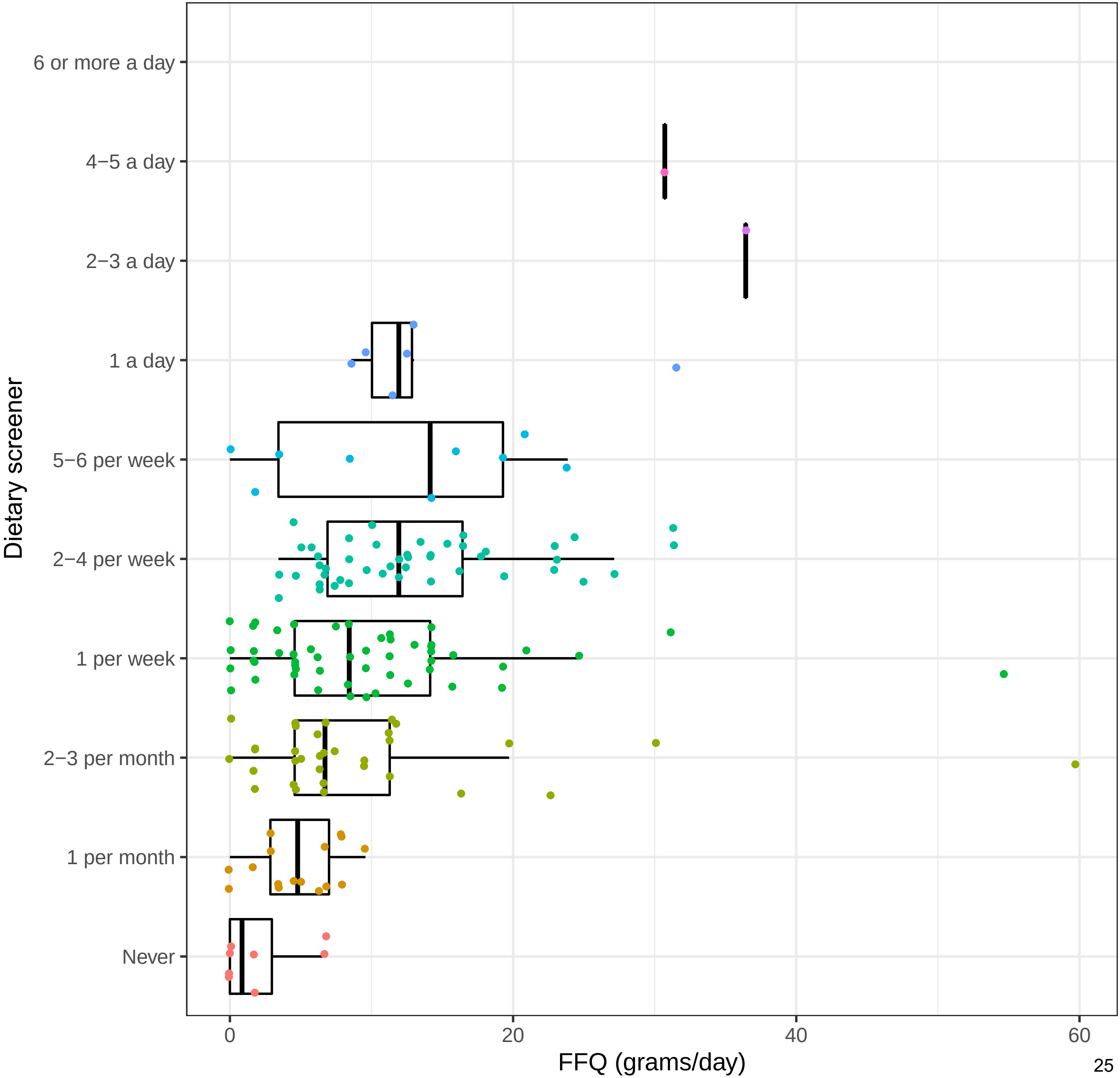

Figure S25. Box and whisker plot for “Waffles/buns/cake/biscuits” in the dietary screener 'MyFoodMonth 1.1' compared to a semi-quantitative food-frequency questionnaire. Participants presented pooled, the box indicating median and interquartile range (25th and 75th percentiles), and whiskers as quartile ±1.5*interquartile range convention.

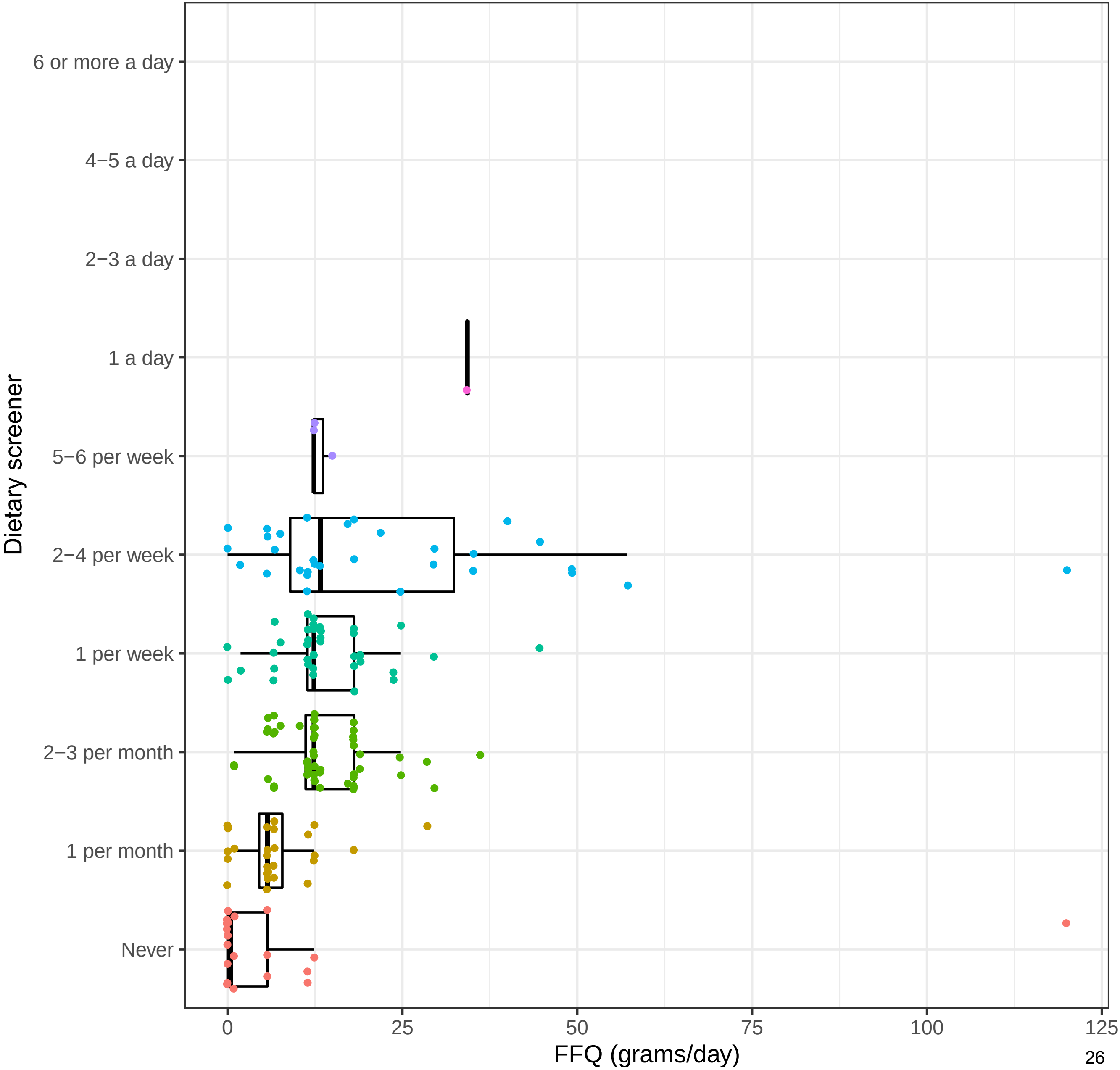

Figure S26. Box and whisker plot for “Ice cream/panna cotta/pudding etc” in the dietary screener 'MyFoodMonth 1.1' compared to a semi-quantitative food-frequency questionnaire. Participants presented pooled, the box indicating median and interquartile range (25th and 75th percentiles), and whiskers as quartile ±1.5*interquartile range convention.

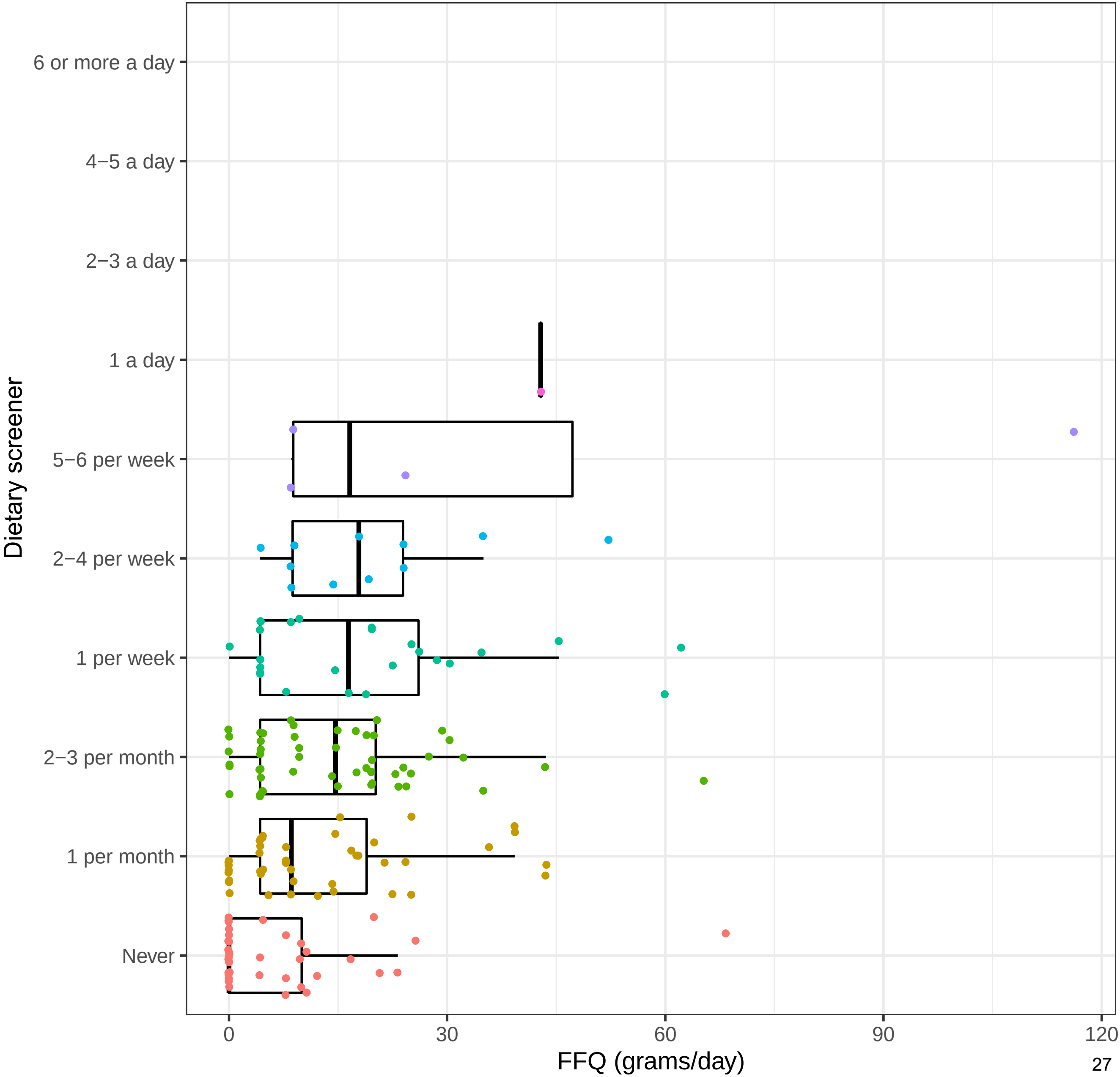

Figure S27. Box and whisker plot for “Sugar-sweetened beverages” in the dietary screener 'MyFoodMonth 1.1' compared to a semi-quantitative food-frequency questionnaire. Participants presented pooled, the box indicating median and interquartile range (25th and 75th percentiles), and whiskers as quartil ±1.5*interquartile range convention.

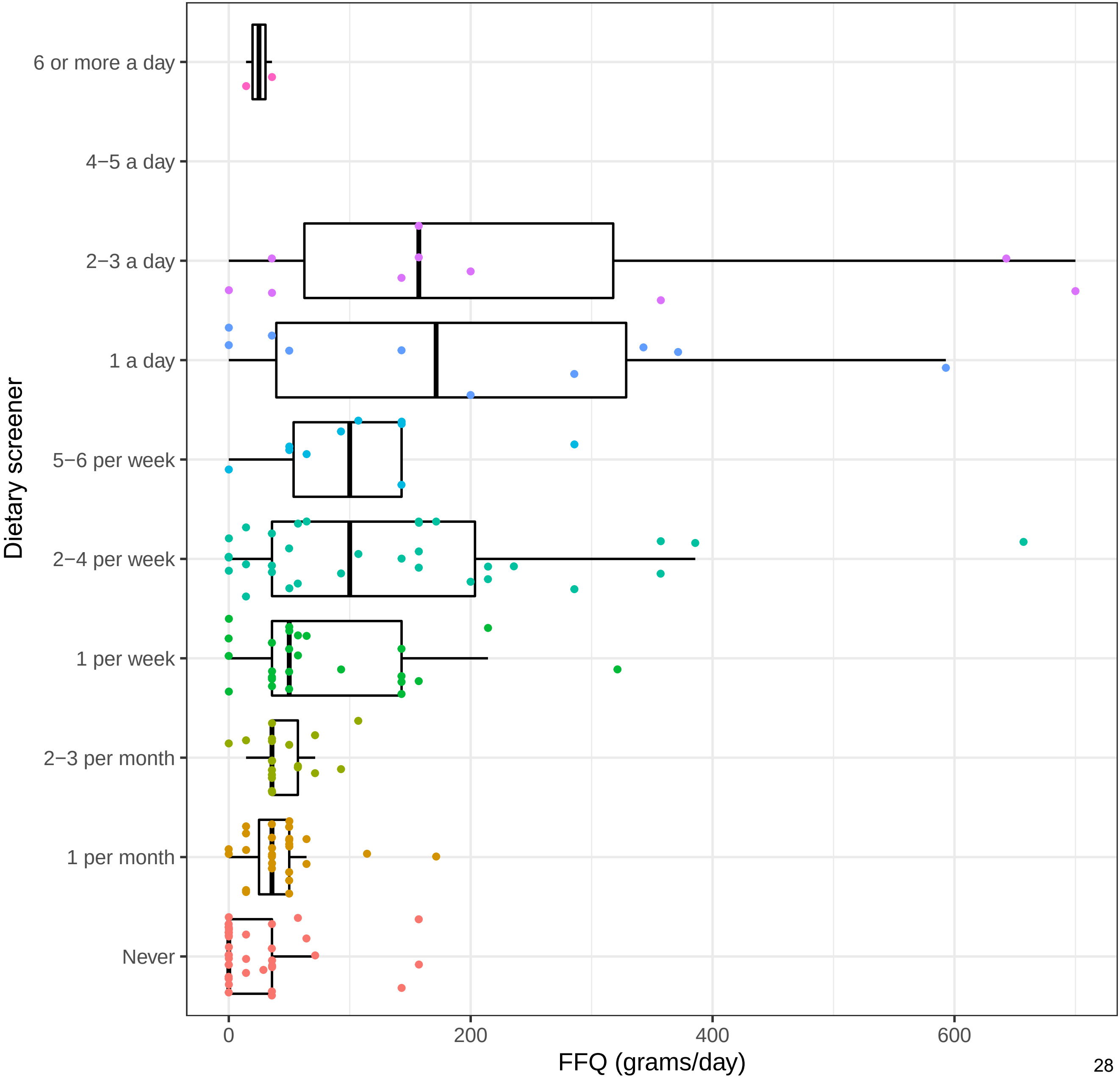

Figure S28. Box and whisker plot for "Energy drinks with sugar" in the dietary screener 'MyFoodMonth 1.1' compared to a semi-quantitative food-frequency questionnaire. Participants presented pooled, the box indicating median and interquartile range (25th and 75th percentiles), and whiskers as quartile ±1.5*interquartile range convention.

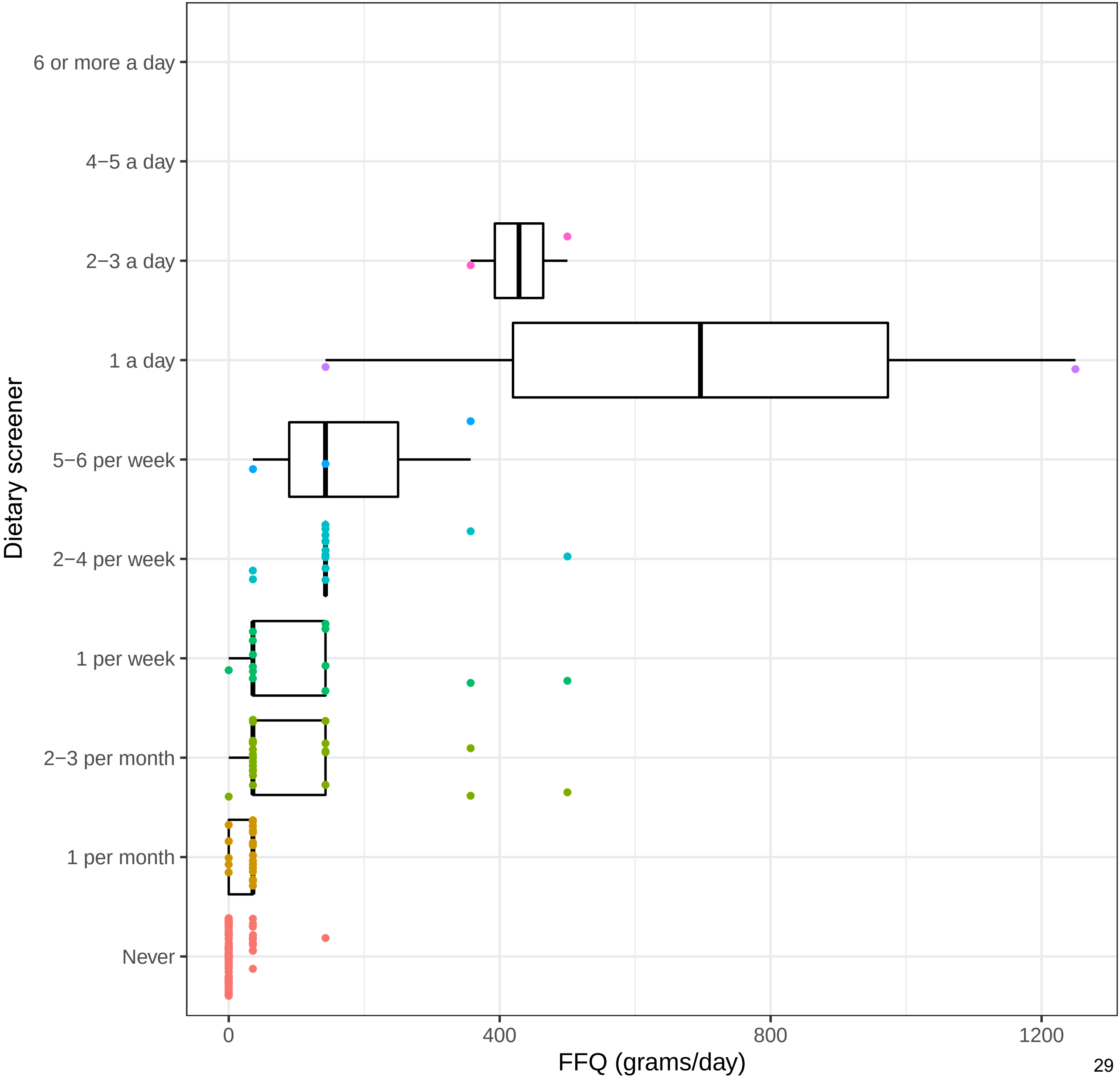

Figure S29. Box and whisker plot for “Coffee/tea with sugar/syrup/honey” in the dietary screener 'MyFoodMonth 1.1' compared to a semi-quantitative food-frequency questionnaire. Participants presented pooled, the box indicating median and interquartile range (25th and 75th percentiles), and whiskers as quartile ±1.5*interquartile range convention.

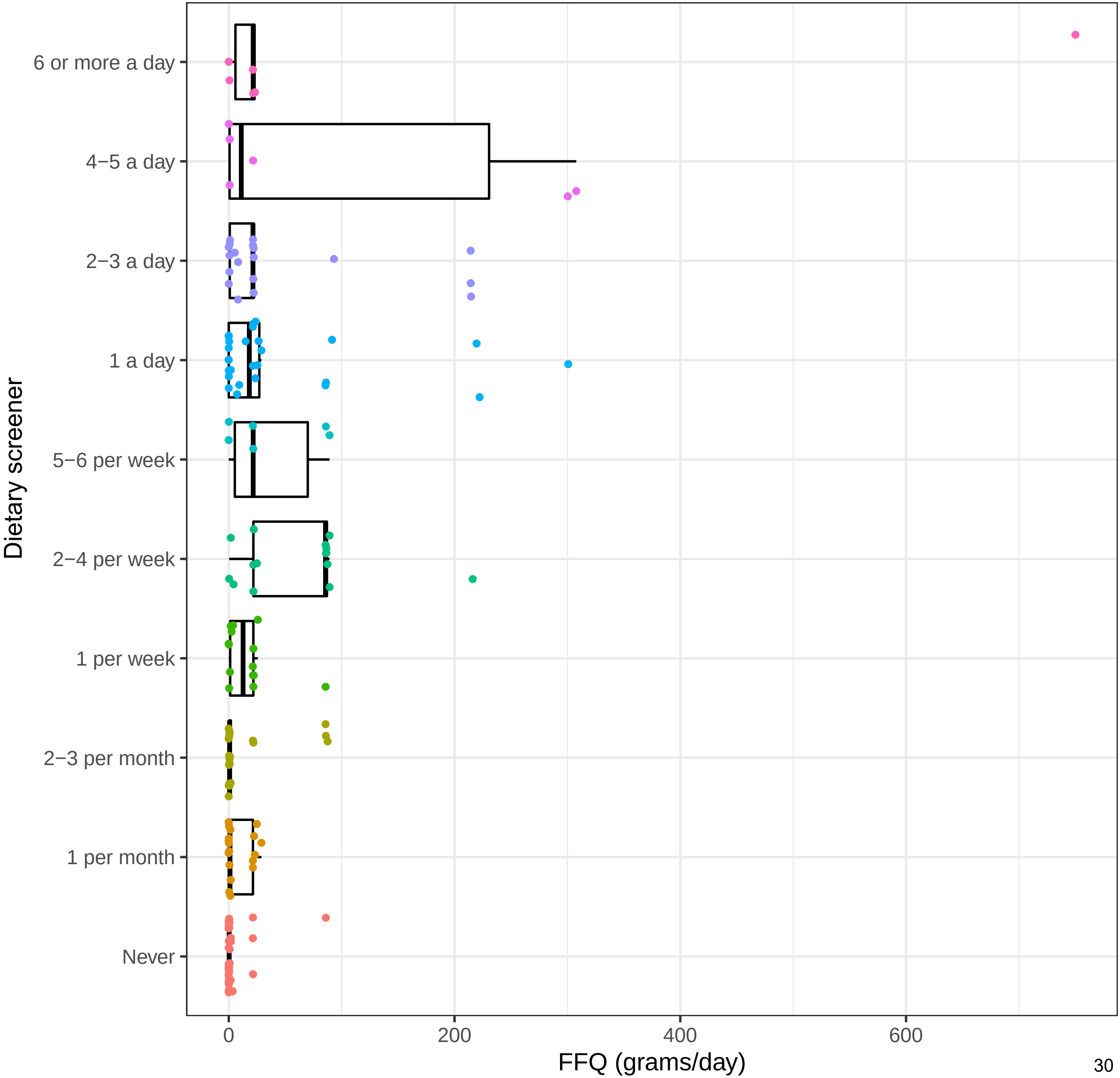

Figure S30. Box and whisker plot for “Alcoholic beverages” in the dietary screener 'MyFoodMonth 1.1' compared to a semi-quantitative food-frequency questionnaire. Participants presented pooled, the box indicating median and interquartile range (25th and 75th percentiles), and whiskers as quartile±1.5*interquartile range convention.

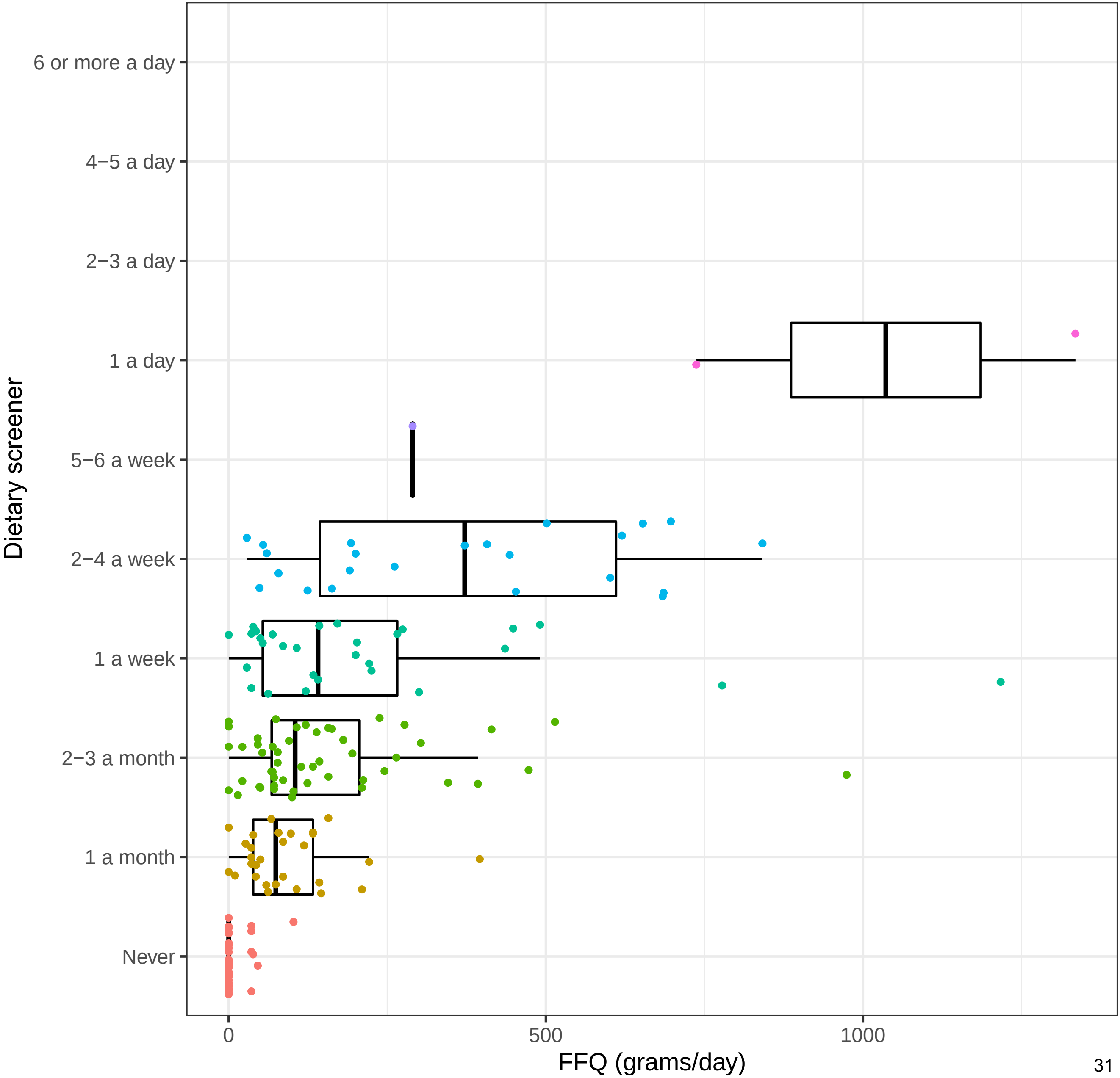

Figure S31. Box and whisker plot for “Water” in the dietary screener 'MyFoodMonth 1.1' compared to a semi-quantitative food-frequency questionnaire. Participants presented pooled, the box indicating median and interquartile range (25th and 75th percentiles), and whiskers as quartile ±1.5*interquartile range convention.

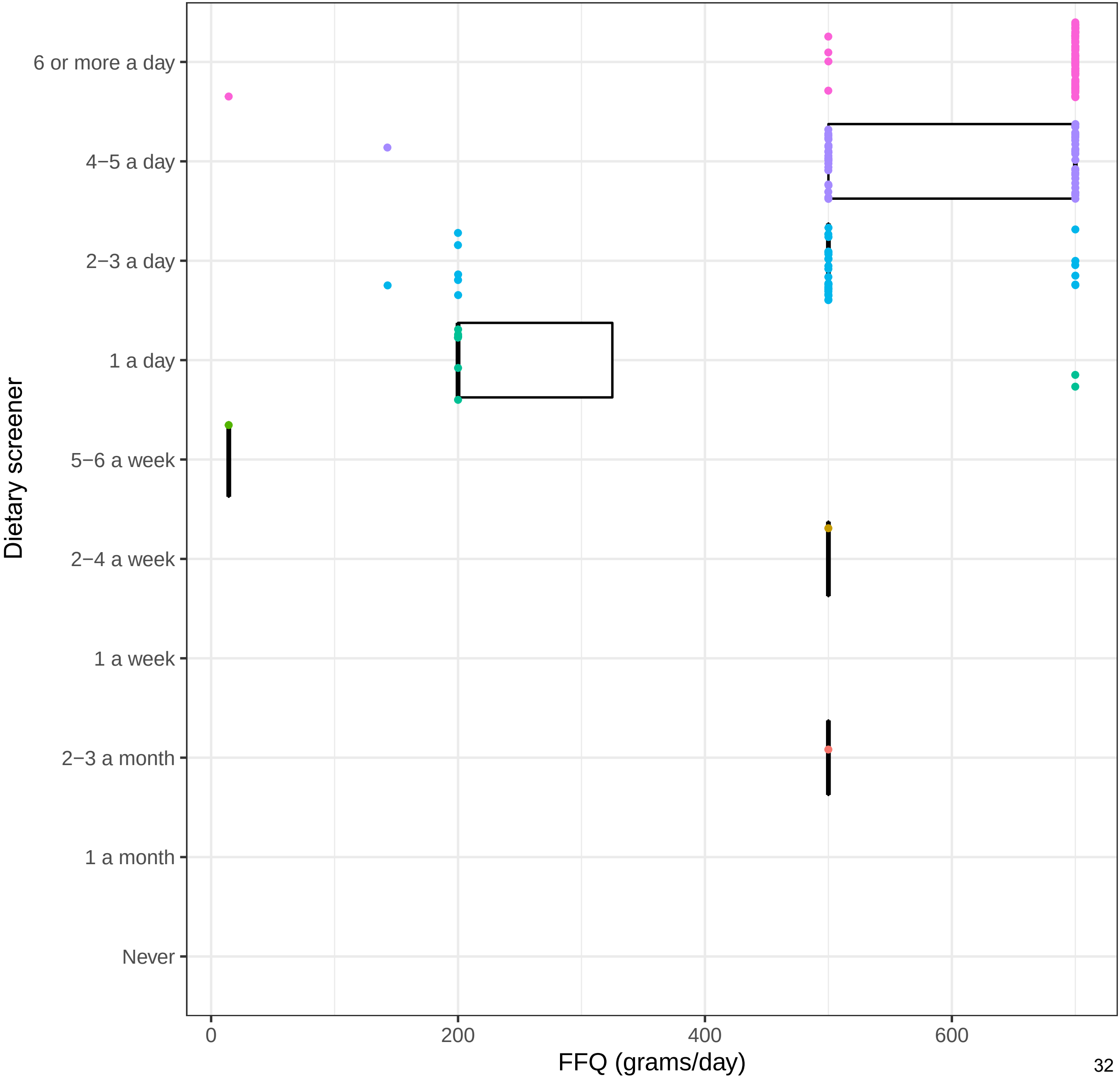

Supplement: Supplementary file 1 [file jnssup.zip › S2048679023000575sup003.pdf]
