# Supplementary material for: Relative validity of a non-quantitative 33-item dietary screener with a semi-quantitative food frequency questionnaire among young adults
Source: J Nutr Sci. 2023 Jul 6;12:e72. doi: 10.1017/jns.2023.57 (PMC10345784; doi:10.1017/jns.2023.57)

Supplementary file 5.

Forest-plots of Kendall's tau-b concordance with 95% confidence intervals for the dietary screener 'MyFoodMonth 1.1' compared to a semi-quantitative food frequency questionnaire, pooled or split by gender.

Figure S32. Forest-plot of Kendall's tau-b correlation coefficient with 95% confidence intervals for 31 food items in the dietary screener 'MyFoodMonth 1.1' compared to a semi-quantitative food frequency questionnaire split by gender.

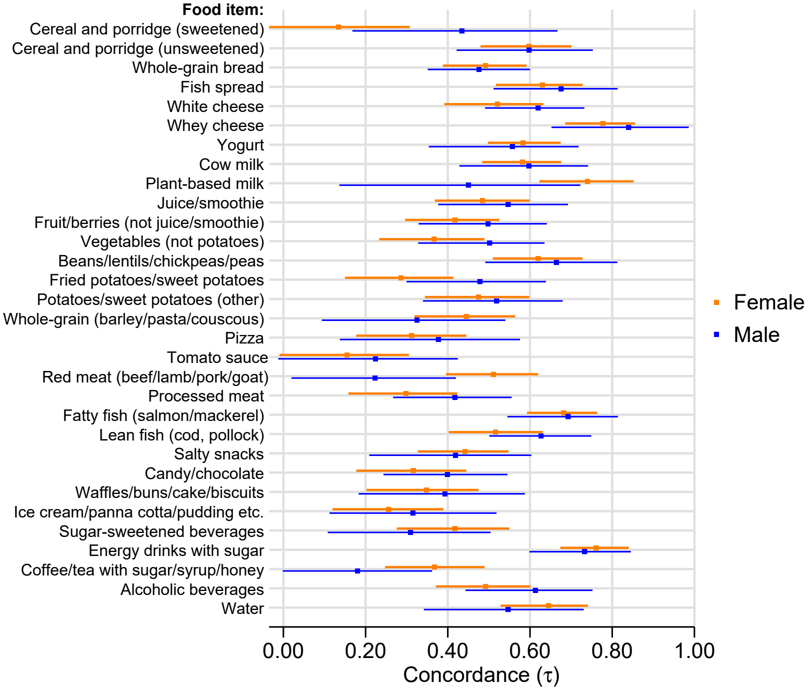

Figure S33. Forest-plot of Kendall’s tau-b correlation coefficient with 95% confidence intervals for single food item ordinal variables not included as aspects of diet quality derived from the dietary screene 'MyFoodMonth 1.1' compared to a semi-quantitative food frequency questionnaire.

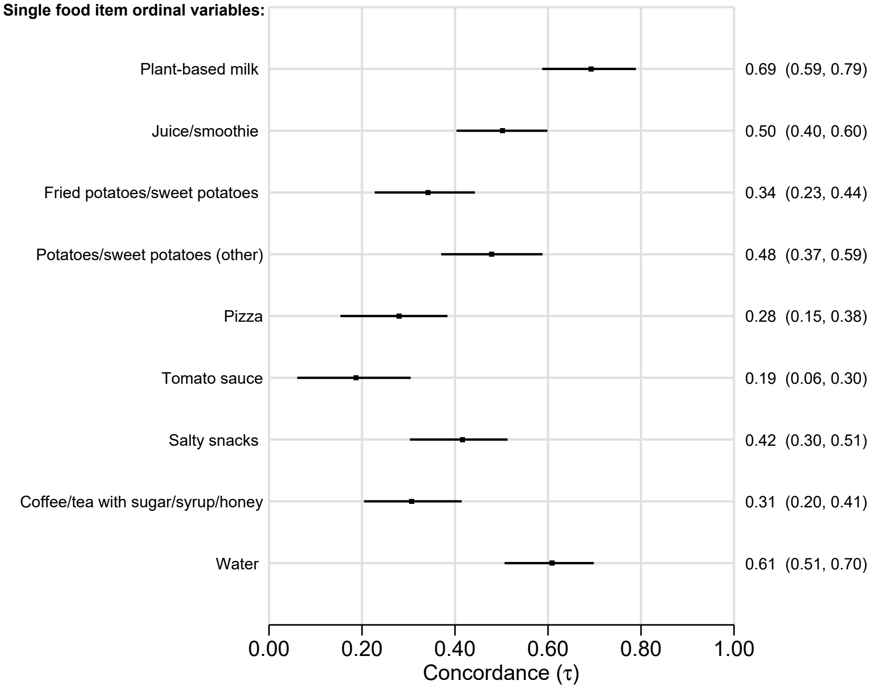

Figure S34. Forest-plot of Kendall's tau-b correlation coefficient with 95% confidence intervals for aspects of diet quality, iodine-rich foods, and calcium-rich foods derived from the dietary screener 'MyFoodMonth 1.1' compared to a semi-quantitative food frequency questionnaire split by gender.

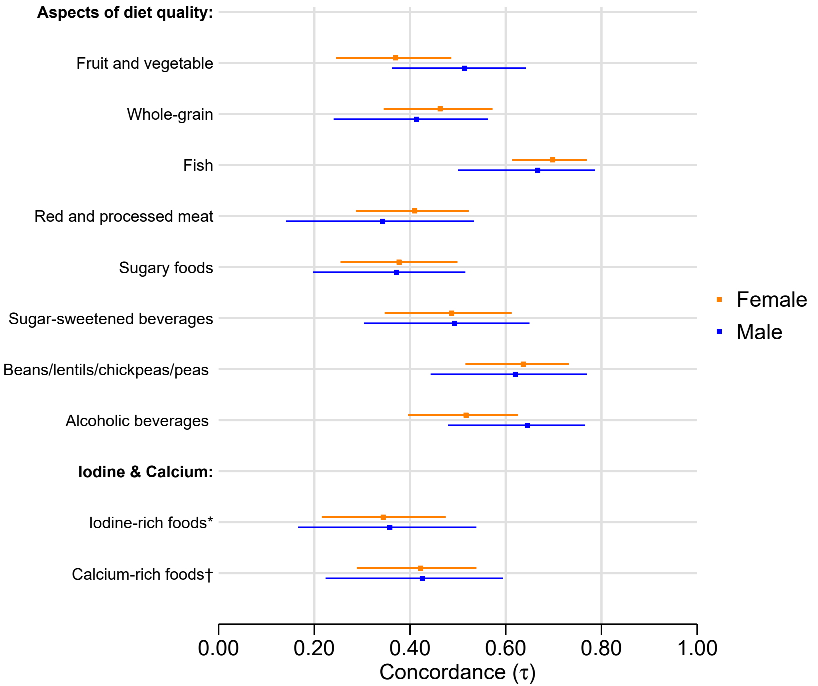

*Compared to iodine intake (µg) per day. †Compared to calcium intake (mg) per day.

Figure S35. Forest-plot of Kendall's tau-b correlation coefficient with 95% confidence intervals for single food item ordinal variables not included as aspects of diet quality derived from the dietary screener 'MyFoodMonth 1.1' compared to a semi-quantitative food frequency questionnaire split by gender.

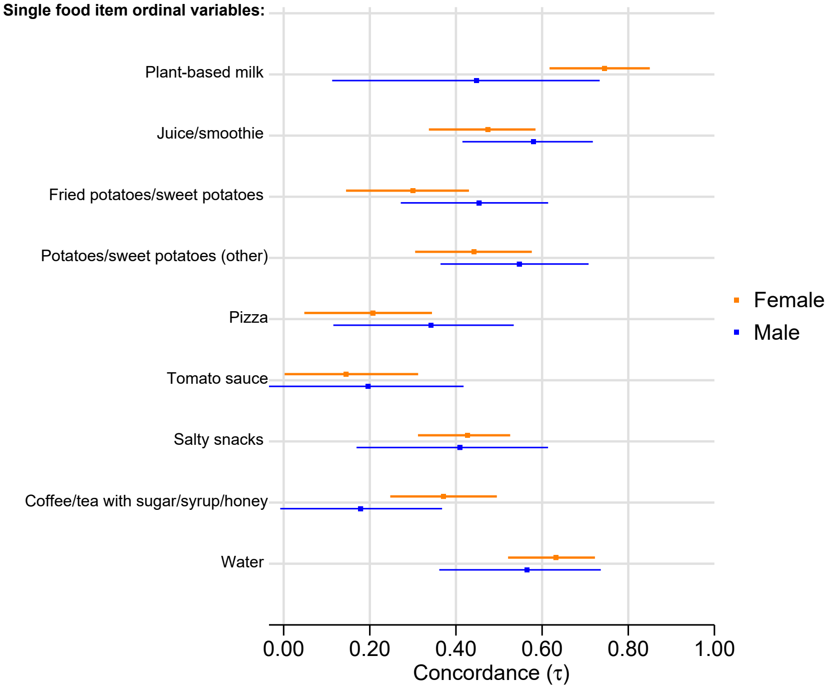

Figure S36. Forest-plot of Kendall's tau-b correlation coefficient with 95% confidence intervals for the diet quality score components derived from the dietary screener 'MyFoodMonth 1.1' compared to a semi-quantitative food frequency questionnaire split by gender.

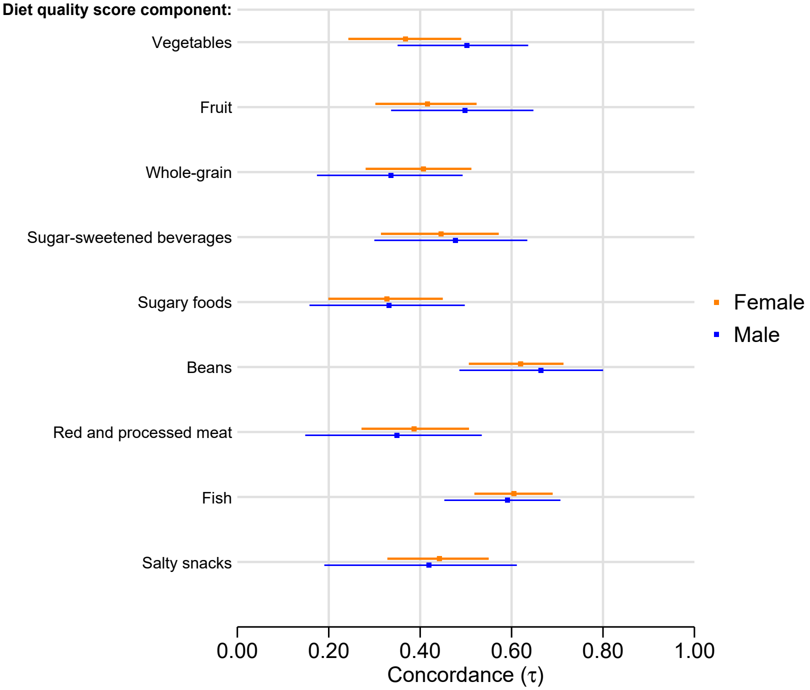

Supplement: Supplementary file 1 [file jnssup.zip › S2048679023000575sup004.pdf]
